# The Heterogeneous Effects of College Education on Outcomes Related to Deaths of Despair

**DOI:** 10.1177/00221465241291845

**Published:** 2024-11-14

**Authors:** Grzegorz Bulczak, Alexi Gugushvili, Jonathan Koltai

**Affiliations:** 1 Gdynia Maritime University, Gdynia, Poland; 2 University of Oslo, Oslo, Norway; 3 Independent researcher

**Keywords:** Add Health, deaths of despair, education, health, inequality

## Abstract

College education features prominently in research on determinants of deaths from substance use disorders and self-harm—outcomes collectively referred to as “deaths of despair” (DoD). Limited attention has been given to whether the protective effects of college education on indicators of despair vary by individuals’ likelihood of college completion. We use data from the National Longitudinal Study of Adolescent to Adult Health for 6,145 individuals to test whether the protective effects of college completion on precursors to DoD vary according to individuals’ propensity to attain a college degree. Understanding whether the benefits of college education differ depending on the propensity to complete it is important for designing effective educational policies. Using the heterogeneous treatment effects approach, we find that individuals with a relatively low propensity for graduating from college but who complete it have a lower likelihood of depressive symptoms, binge drinking, prescription drug abuse, and hard drug use.

“Deaths of despair” (DoD)—mortality linked to suicide, drug overdose, and alcohol-induced liver disease—have increased substantially since the turn of the twenty-first century (Case and Deaton 2015, 2020). Recent literature characterizes this surge as a socially patterned epidemic (King, Scheiring, and Nosrati 2022). Case and Deaton (2022) highlight a “Great Divide” in these trends, with rates of DoD increasing predominantly among non-Hispanic White individuals lacking a four-year college education. Conversely, rates of DoD have stagnated or diminished for Americans possessing a bachelor’s degree or higher. One prevailing explanatory narrative is an implied causal chain linking deteriorating social and economic conditions to an increase in the lived experience of despair among individuals with lower levels of education, whereas those with a college degree are shielded from many of these risk factors (Fishman and Gutin 2021; Gaydosh et al. 2019; Shanahan et al. 2019).

Two issues complicate the interpretation of degree attainment as protective against despair-related outcomes. First, despite the prevalence of the college-for-all narratives and the convergence of college aspirations among individuals with advantaged and disadvantaged socioeconomic origins, significant inequalities in enrollment and completion persist in the United States (Silva and Snellman 2018). These patterns raise concerns about selection bias because early life disadvantages can set in motion a chain of adversity that stifles educational attainment and compromises health and well-being (Montez and Hayward 2014; Schafer, Wilkinson, and Ferraro 2013). Second, emerging literature suggests that the effects of education may vary according to individuals’ propensities to complete college. Recent theoretical and methodological developments highlight that individuals may differ not only in their likelihood to attain a college degree but also in how the degree’s effects manifest based on their propensity to attain it (Brand et al. 2019; Brand and Xie 2010; Cheng et al. 2021; Schafer et al. 2013; Xie et al. 2020).

Here, we investigate whether the protective effects of college completion on depressive symptoms and other indicators of despair vary according to childhood disadvantage and an individual’s propensity to attain a college degree. This approach makes an important contribution to understanding the nature of the association between education and despair-related outcomes by mitigating the issue of selection bias and considering who benefits most from college degree completion.

Understanding whether the benefits of college education differ depending on the propensity to complete it is important for several reasons. If individuals with a lower likelihood of completing college derive more significant benefits from obtaining a degree, this insight could inform the design of educational policies and interventions. For instance, it could support the expansion of higher education initiatives, such as free community college or targeted financial aid, to ensure that these programs effectively reach and benefit those most at risk of not attending or completing college. By focusing resources on students who are less likely to complete college but stand to gain the most from it, policymakers can better allocate public funds to maximize socioeconomic returns. This approach aligns with broader goals of reducing inequality and enhancing social mobility because those who overcome significant barriers to complete college may experience notable improvements in health and well-being outcomes, thereby reducing overall societal costs associated with adverse outcomes such as substance abuse and mental health issues.

## Background

Education is widely recognized as a key determinant of health outcomes. According to the social determinants of health perspective, education influences health by shaping access to resources, social support, and opportunities for healthy behaviors (Braveman, Egerter, and Mockenhaupt 2011; Marmot 2005). Additionally, the fundamental cause theory posits that education serves as a fundamental cause of health disparities because it provides individuals with flexible resources—such as knowledge, money, power, prestige, and social connections—that can be used to avoid risks and adopt protective strategies (Phelan, Link, and Tehranifar 2010). These frameworks help explain why educational attainment is consistently linked to a wide range of health outcomes, including those related to despair.

Despite a rapidly growing literature, very little is known about the concrete mechanisms linking education to indicators of despair at the individual level (Case and Deaton 2022; Gutin et al. 2023). One major barrier to mapping these mechanisms is the ambiguous usage of the term “despair.” Although the term often implies elements of psychological suffering, physical pain, and self-inflicted harm, these states and behaviors are rarely operationalized or measured in the DoD literature (Case and Deaton 2022; Gaydosh et al. 2019; Gutin et al. 2023). Shanahan et al. (2019) advocate for the use of extant longitudinal data sets that feature repeated measures of despair. These authors further argue for more research focusing on protective factors that could shield individuals so that preventions and interventions might have an empirical basis. Following these recommendations, Fishman and Gutin (2021) draw on high-quality panel data and repeated measures to estimate the association between educational attainment and four indicators of DoD: drug use, painkiller use, frequent binge drinking, and suicidal ideation. Consistent with patterns found in the broader DoD literature, the authors found that educational attainment reduces the probability of despair outcomes.

Fishman and Gutin’s (2021) study is informative, but two key issues still complicate our understanding of the association between educational attainment and despair outcomes. First, although life course transitions such as college degree completion shape future psychological and behavioral outcomes, educational attainment is also influenced by antecedent conditions (Elder, Johnson, and Crosnoe 2003; Schafer et al. 2013). For example, low household socioeconomic position, neighborhood disadvantage, and traumatic childhood experiences set in motion a chain of adversity that can stifle educational attainment (Montez and Hayward 2014; Wightman and Danziger 2014; Wodtke, Harding, and Elwert 2011). Because early life adversity is also associated with trajectories of poor mental health through midlife (Desch et al. 2023), studies that do not comprehensively account for these pathways may identify a spurious relationship between education and indicators of DoD.

Second, if educational attainment indeed shields individuals from despair-related outcomes, a critical question concerns who benefits most from a college degree. Recent theoretical and methodological developments have shed light on the possibility that individuals may not only differ in their propensity to attain a college degree but that the effects of a college degree may also depend on an individual’s propensity to attain this degree in the first place (Brand et al. 2019; Brand and Xie 2010; Cheng et al. 2021; Xie et al. 2020). For example, studies have shown that individuals who were least likely to graduate from college benefited the most from its protective effects on depression in early adulthood (Bauldry 2015) and cardiovascular health at ages 25 to 74 (Schafer et al. 2013). We now consider the theoretical reasons for expecting heterogeneity in the effects of college education on DoD-related outcomes.

### Resource Multiplication, Resource Substitution, and DoD-Related Outcomes

The recent emphasis on positive implications of intergenerational upward mobility in social stratification literature is in line with the growing realization that individuals are different not only in their sociodemographic and socioeconomic characteristics but also by systematic variation in particular treatment effects by the propensity for being treated, in the case of this study—having a college degree (Xie 2013; Xie, Brand, and Jann 2012). So far, the heterogeneous consequences of tertiary educational attainment have been explored in relation to cognitive/noncognitive skills, divorce, fertility, civic participation, voting behavior, and economic outcomes, such as the level of wages (Ahearn, Brand, and Zhou 2023; Brand 2010; Brand and Davis 2011; Brand et al. 2019; Brand and Xie 2010; Cheng et al. 2021; Xie et al. 2020). These studies usually find that college education’s social and economic consequences are positive among individuals with comparatively disadvantaged social backgrounds. To our knowledge, apart from a few exceptions (Bauldry 2015; Schafer et al. 2013), no studies have investigated the heterogeneous links between college education as a treatment and indicators related to DoD.

The role of education has been extensively investigated in relation to DoD outcomes, and a recent scoping review suggests that educational attainment is indeed one of the most critical social determinants of DoD in the United States (Beseran et al. 2022; Case and Deaton 2020). Studies indicate that education might have a different association with specific causes of DoD. For instance, educational inequalities appear to be more important for opioid-related deaths than for suicides or alcohol-related deaths (Geronimus et al. 2019). There is also an ongoing debate about whether low education leads to higher despair or if tertiary education decreases DoD outcomes (Zeglin, Niemela, and Baynard 2019). Nonetheless, almost no scholarly attention has been paid to whether the effect of college education on DoD varies by the specific trajectories of how individuals attained their education.

An interrelated set of theoretical approaches, often referred to as “cumulative advantage,” “resource multiplication,” or “multiplier interplay” (Ross and Mirowsky 2011; Veenstra and Vanzella-Yang 2022), predicts that a college degree would have a more substantial effect on DoD outcomes for those individuals with advantaged social origins because the attained education in adulthood facilitates expression of positive effects of multiple components of earlier life course socioeconomic position. For instance, a college degree helps individuals use their earnings or wealth in ways conducive to health, such as obtaining comprehensive health insurance, understanding the positive implications of more expensive healthy diets, or living in neighborhoods free of various forms of pollution. The described theoretical approach, however, received only limited empirical support with specific measures of well-being, such as self-rated health (Andersson 2016).

An alternative theoretical perspective based on the life course approach implies that individuals experience various life events and spend time in different socioeconomic environments. These exposures accumulate and can manifest themselves in the nexus between a college degree and DoD outcomes (Ragnarsdottir et al. 2017). The potential mechanisms linking education and health, such as individuals’ self-esteem and lifestyle, might have varying effects depending on the pathways of attaining tertiary education. Sociological and social psychological theories on the well-being implication of intergenerational mobility, such as the “rising from the rags” perspective, suggest that moving upward in the social hierarchy after overcoming various barriers can be associated with an improved psychological mindset, which is, in turn, linked negatively to depressive symptoms and positively to psychological well-being (Gugushvili, Zhao, and Bukodi 2019). Therefore, the protective effect of college completion on indicators of despair will likely vary by an individual’s social origin characteristics (Schafer et al. 2013).

More specifically, intergenerational upward mobility can often result in boosting locus of control, having confidence in overcoming difficulties in life, and developing a sense of gratitude to the existing environment that enabled upward mobility, and these positive effects might be more vital for individuals who were especially deprived during their childhoods (Day and Fiske 2017; Schafer et al. 2013). The described processes can be called the “resource substitution” mechanism. It implies that tertiary educational attainment among those who are least likely to complete college compensates for preexisting disadvantages (Bauldry 2015; Koltai and Schieman 2015). This theoretical perspective is supported by evidence that well-educated individuals develop a sense of influence over their lives and usually engage in healthier behaviors (Gugushvili et al., 2019; Gugushvili, Zhao, and Bukodi 2020; Ross and Mirowsky 2011). Attainment of a college degree can also expand a social network, which can have beneficial consequences related to DoD outcomes. Furthermore, moving up in the social hierarchy against the odds may also reflect a type of individual who is more resilient to despair triggers (Mackenbach 2020).

A supplementary explanation of DoD outcomes related to college completion can be the reference group theory (Hyman 1942; Merton and Rossi 1957). According to this perspective, individuals tend to evaluate their life situations based on their expectations when they were younger. This can involve comparing themselves to their parents or their high school peers (Gugushvili 2021). In the context of the present study, if someone expected to complete college and actually did so, they may not derive as much benefit from that experience as someone who did not expect to complete college due to childhood disadvantages but still managed to attain a college degree. In the latter case, individuals’ achievement surpasses their expectations, and they might feel that their lives turn out better than expected. Furthermore, a cross-national European study found that those who compared themselves with their parents were less likely to report good health compared with those who did not compare their own economic situation with any specific reference group (Gugushvili, Jarosz, and McKee 2019).

### Heterogeneous Effects on DoD by Gender and Race-Ethnicity

There are also reasons to believe that the heterogeneous consequences of college education might vary by individuals’ gender and race-ethnicity. For instance, some evidence suggests that for later life outcomes such as depressive symptoms and smoking, childhood and adolescence advantages and disadvantages matter more for females than for males, while upward mobility (in our case, by completing college) implications are more salient for males than females (Gugushvili, Zhao, and Bukodi 2019). According to the theory of causal attribution, males and females might differ in their understanding and assessment of the causes behind their success in life (Miller and Ross 1975). Due to either inherent psychological differences or different modes of socialization, females may be less likely than males to attribute a college completion to their own merits, abilities, and effort (Meece, Glienke, and Burg 2006). Dow et al. (2019) indeed find heterogeneous effects by gender concerning the effect of minimum wage on DoD outcomes. According to the assumptions described, disadvantaged males may benefit more psychologically from upward social mobility through obtaining a college degree than females.

Another dimension by which heterogeneous effects of college completion on DoD-related outcomes might vary in the United States is individuals’ race-ethnicity. Growing evidence indicates how structural racism via myriad mechanisms contributes to racial-ethnic inequalities in morbidity and mortality outcomes (Bailey et al. 2017; Hankerson et al. 2022). In addition, as the study by Gaydosh et al. (2018) suggests, the implications of college completion on DoD-related outcomes might be determined by varying levels of stressors experienced by different racial-ethnic groups. According to the John Henryism hypothesis, high effort associated with enduring constant psychosocial stressors among disadvantaged racial-ethnic groups without sufficient socioeconomic resources could lead to detrimental DoD outcomes (Bennett et al. 2004; Zilioli et al. 2022). A related body of evidence also indicates that disadvantaged Black Americans who overcome various life constraints might benefit psychologically but still experience more significant physiological dysregulation (Brody et al. 2013; Miller et al. 2015). Cherlin (2016) elaborated on the importance of the reference group theory in explaining the racial differences in DoD outcomes given that White individuals without college degrees likely remember parents who were sustained by the booming industrial economy of postwar America. Along with this prediction, Zhou and Pan (2023) find that college completion has a strong equalizing effect on earnings between Black and White males.

Overall, our study contributes to the existing literature by exploring the specific total, gender, and race-ethnicity heterogeneous effects of education on DoD-related outcomes by explicitly considering individuals’ likelihood of attaining a college degree in the United States. We overcome prior gaps in the literature by drawing on Brand and Xie’s (2010) treatment effect approach to assess the relationship between college attainment and five outcome measures related to DoD. We study heterogeneous effects from a long-term perspective by using information from three waves, each approximately eight years apart, of high-quality panel survey data. Crucially, we exploit an extensive range of early life conditions in our modeling strategy, allowing us to investigate whether the college advantage for midlife despair is conditional on the propensity to attain a college degree. To our knowledge, this study is the first that simultaneously investigates the heterogeneous effects of educational attainment on various outcomes associated with DoD and also accounts for potential health selection effects and a wide array of childhood characteristics that predict college completion.

## Data and Methods

### Data Set

We based our analyses on data from Wave I (1994–1995), Wave IV (2008), and Wave V (2016–18) of the National Longitudinal Study of Adolescent to Adult Health (Add Health) collected in the United States (Harris et al. 2019).^1^ Add Health contains information describing childhood, adolescence, and adulthood sociodemographic, socioeconomic, and health characteristics. Numerous past studies have used this data set to explore the determinants of educational attainment and the links between education and health-related outcomes (e.g., Assini-Meytin et al. 2022; Bauldry 2015; Fletcher 2008). Our analysis was restricted to 6,145 individuals who participated in Wave V of the survey (response rate = 69%) and who also had no missing information on the variables of interest in Waves I and IV. The average age of respondents at the time of the Wave V data collection was 37 (range = 32–42). Most individuals have completed their education at this age, and the prevalence of outcomes related to DoD is already high (Kaplan et al. 2014). For descriptive statistics for the employed variables, see Table S1 in the online version of the article.

### Outcome Measures Linked to DoD

We focused our analysis on five outcome measures related to DoD (Wave V)—depressive symptoms, binge drinking, misuse of painkillers, hard drug consumption, and suicidal thoughts. First, our mental health measure was based on the short version of the Center for Epidemiologic Studies Depression Scale (CES-D; Radloff 1977). The following four questions were used to construct the CES-D scale: “How often was the following true during the past week? You felt (1) depressed; (2) sad; (3) could not shake off the blues even with help from my family or friends; (4) happy.” We inverted the responses to the last question so that higher scores indicated greater depressive symptoms. In each case, 1 indicated “never,” and 4 indicated “most of the time”; a higher total score indicated poorer mental health. We constructed a binary indicator equal to 1 if the total score was above 8 and equal to 0 otherwise. This indicator will likely identify individuals with moderate to severe depression (Fu, Si, and Guo 2022; Lewinsohn et al. 1997; Williams, Li, and Hay 2020).

Our second measure, binge drinking, was a binary indicator that equaled 1 if the respondent reported having four (females) or five (males) or more drinks in response to the following question: “Think of all the times you have had a drink during the past 30 days. How many drinks did you usually have each time? A ‘drink’ is a glass of wine, a can or bottle of beer, a wine cooler, a shot glass of liquor, or a mixed drink.” For our fourth measure, painkillers use was a binary indicator that equaled 1 if the respondent in the past 30 days had taken certain prescription drugs, such as Vicodin, OxyContin, Percocet, Demerol, Percodan, or Tylenol with codeine, that were not prescribed for them or had taken the prescribed drug more often than prescribed or for more extended periods than prescribed. Fourth, hard drugs use was a binary indicator that equaled 1 if in the past 30 days, the respondent used any of the following drugs: cocaine (crack, coca leaves), crystal meth, heroin, or other types of illegal drugs, such as LSD, PCP, ecstasy, or mushrooms or inhalants. Fifth, the binary indicator for suicidal thoughts was equal to 1 if the respondent seriously thought about committing suicide in the past 12 months.

### College Completion and Treatment Selection Variables

Our primary variable of interest as the treatment, completed college education, was based on Wave V educational attainment. We created a binary variable that indicated completion of four-year college or bachelor studies. We also checked how alternative measures, such as attaining some college education or postgraduate degree, and Wave IV educational attainment were associated with DoD-related outcomes (see Supplemental Material in the online version of the article).

On top of the individuals’ sociodemographic characteristics such as age, gender, race-ethnicity, and Peabody test scores (a proxy measure of the level of IQ), we identified three sets of variables that were likely to affect the chances of completing college in the United States. These sets included Wave I data on (1) parents, (2) neighborhoods, and (3) schools and Wave IV retrospective information on further early life disadvantages.

Parental characteristics included Wave I information about educational and occupational attainment and household income. For educational attainment, we relied on the highest reported education by either of the parents (10 categories). Based on these data, we created quintiles and constructed a binary measure equal to 1 if parental education was in the lowest quintile. Similarly, we used the highest level of occupation obtained by the parents (10 categories) to create 5 occupational categories. We took the average Nam-Power-Boyd scale score for each of the 10 groups and constructed 5, where no occupation = 1 and Nam-Power-Boyd scale score from 70 to 100 = 5 (Gugushvili and Bulczak 2023; Nam and Boyd 2004; Ueno, Peña-Talamantes, and Roach 2013). Subsequently, we divided the original household’s income variable into quintiles after accounting for household size based on equivalence scales: income / (household size)^.5^ (Bulczak and Gugushvili. 2022; Johnson, Smeeding, and Torrey 2005). In addition to parents’ marital status, we considered indicators for single parenthood at ages 0 and 13. Parental variables also included binary indicators equal to 1 if the household received welfare or had no health insurance and parents binge drank, talked about separation, fought, or were in jail.

Based on past studies, we controlled for the following neighborhood characteristics: low proportion of White individuals in the block where individuals resided (bottom 20%), low median income (bottom 20%), and high unemployment (top 20%; Garner and Raudenbush 1991; Levy 2019). Last but not least, we accounted for schools’ characteristics by including indicators equal to 1 if, based on the schools’ administrator report, the school had an average class size above 30, a low proportion of teachers with a master’s degree, a high proportion of students held back, a high dropout rate, and a low attendance rate.

### Additional Childhood Adversity Measures

We followed previous studies in the field of childhood adversities and identified a set of additional measures that were likely to affect the chances of college completion (Bauldry 2015; Gaydosh et al. 2018). Based on respondents’ answers recorded during Wave I, we constructed dummy variables, each equal to 1, if the respondent reported running away from home or that someone pulled a knife or gun on them during the past 12 months. We introduced a poor parental care indicator equal to 1 if the respondent reported that parents cared about them somewhat, very little, or not at all.

Abuse during childhood and adolescence was represented by two binary variables equal to 1 if respondents retrospectively (in Wave IV) reported that before their 18th birthday, a parent or other adult caregiver touched them in a sexual way, was forced to touch them in a sexual way, was forced to have sexual relations, and hit them with a fist, kicked, or threw them down on the floor, into a wall, or downstairs. This retrospective dimension is likely to improve the victimization measurement precision, as past research indicates (Aalsma et al. 2002). During Wave I home visits, interviewers were asked questions about the respondent’s household condition, surroundings, and the respondent. Based on these assessments, we introduced the following binary measures, equal to 1, if the interviewer noticed any evidence of drinking in the household, was afraid about their own safety, noticed that the building in which the respondent lived was in poor condition, or observed that the respondent was poorly groomed.

### Accounting for the Initial Values of Outcomes Related to DoD

Past research shows that the problem of health selection is likely to be important in the case of educational attainment—initial health affecting both educational and later life health outcomes (Bulczak, Gugushvili, and Zelinska, 2022; Gugushvili et al. 2021; Haas 2006; Kröger, Pakpahan, and Hoffmann 2015). To address this issue, we accounted for the corresponding Wave I DoD measures for each of the five considered DoD-related outcomes in our empirical estimates. For example, in the CES-D (Wave V) outcome, we used the exact Wave I CES-D match in the health selection model.

### Statistical Analyses

For our empirical analyses, we used a strategy often referred to as heterogeneous treatment effects (HTEs) proposed by, among others, Brand and Xie (2010). This analytical strategy implies using binary (probit, in our case) regressions to predict the probability of completing college and derive propensity scores for each individual in the sample. The generated propensity scores are used to balance the distributions of the covariates between college graduates and non-college graduates, followed by the estimation of the heterogeneous results along with the continuum of propensity scores (Brand, Pfeffer, and Goldrick-Rab 2014).

Out of several approaches, our HTE choice relied on the smoothing-differencing (SD) method, but we also tested the robustness of our main results using an alternative method, the stratification-multilevel HTE (Xie et al. 2012). The advantage of the SD method over other alternatives is that it involves a single modeling procedure and allows the observation of nonlinear HTEs of education on outcomes related to DoD, which we expected to occur in our case (Choi and Park 2016). Brand and Xie (2010) provide a detailed discussion about the advantages and disadvantages of different HTE approaches, including the stratification-multilevel method. Our estimation strategy involved the following steps. First, we used probit regression to estimate the propensity score for completing college:

$$P(D_{i}=1|X_{i})=\Phi (X_{i}\beta )$$

where Φ is the cumulative distribution function of the standard normal distribution, *D_i_* is a binary indicator of college completion, *X_i_* is a vector of covariates, and β is a vector of coefficients.

A valid estimate of the propensity score is required to move to the next stage. This is referred to as the “ignorability assumption,” which states that after controlling for a set of pretreatment covariates, no other confounders separate treated and untreated individuals. Based on previous studies, the extensive set of covariates discussed earlier is likely to meet this requirement (Brand and Xie 2010; Choi 2018). Although the ignorability assumption cannot be directly tested, we implemented several robustness checks to support its validity. We verified the balance of covariates between the treated and control groups using standardized mean differences. This ensured that the covariates were balanced within the propensity score strata. We conducted sensitivity analyses, including Rosenbaum bounds, to evaluate how unmeasured confounding might impact our results. We also estimated logit models and took into account recent improvements in propensity score specifications by implementing the iterative propensity score logistic regression (Itpscore) approach as an alternative model search procedure (Imbens and Rubin 2015; Moore, Brand, and Shinkre 2021). More specifically, we considered alternative specifications of the propensity score model, including interactions and higher-order terms. The Itpscore algorithm identifies the most suitable propensity model by selecting covariates and their interactions that give the largest gains in the logit log-likelihood function. These approaches yielded consistent results, and we present the final specification with all linear regressors.

After the first step, for the control and the treatment groups, separate nonparametric regressions of the outcome variable on the propensity scores were fitted (the smoothing step). The outcome *Y_i_* was modeled as a function of the propensity score:

$$Y_{i}=\alpha +\delta D_{i}+f({\hat{P}}_{i})+\epsilon_{i}$$

where ${\hat{P}}_{i}$ is the estimated propensity score, *f* is a smoothing function, α is an intercept, δ is the treatment effect, and ε_*i*_ is the error term. To estimate HTEs, we conducted separate nonparametric regressions for the treatment and control groups:

$$\begin{array}{l} {\hat{Y}}_{1}\;(P_{i})=g_{1}(P_{i}\;)+\epsilon_{1i}\\ {\hat{Y}}_{0}\;(P_{i})=g_{0}(P_{i})+\epsilon_{0i}, \end{array}$$

where *g*_1_ and *g*_0_ are nonparametric regression functions for the treatment and control groups, respectively. The actual HTE was derived by the difference in the nonparametric regression functions:

$$\Delta (P_{i}\;)={\hat{Y}}_{1}\;(P_{i}\;)-{\hat{Y}}_{0}\;(P_{i}).$$

This step used a local polynomial regression as a smoothing device with degree 1 Epanechnikov kernel and bandwidth 0.2. Changing the bandwidth did not affect the estimates noticeably. We restricted the evaluation grid to the common support of the propensity score in the treatment group and control groups. We used at least 50 observations as the default number of points at which the smooth was calculated. Changes to this evaluation procedure did not affect the main estimates noticeably; most visible changes can be observed on the edges of the estimated propensity score with larger confidence intervals.

## Results

### Treatment Selection Estimates

We start describing the results of the analyses for obtaining propensity scores using models that predict the probability of completing college in the United States. We used a rich set of variables related to the Add Health participants’ early lives that are likely to influence their chances of graduating from college. The results of these estimates from probit regression models, presented in Table 1, indicate the key predictors of college completion. Expectedly, gender and race are identified as important determinants. The odds of finishing college increase with the Peabody test score, parental education, parental occupation, and parental income. Growing up with a single parent as a child and in a household receiving welfare support is associated with lower chances of completing college. Several school characteristics also matter, particularly a high proportion of students held back and a high dropout rate. Furthermore, childhood adversity measures such as poor dwelling conditions, having a parent in jail, having a knife pulled at the respondent, and running away from home significantly reduce the chances of completing college. We also find evidence on health selection given that depressive symptoms, binge drinking, misuse of painkillers, and hard drug consumption at Wave I are negatively linked to college completion.

**Table 1.** Probit Regression Estimates Predicting College Completion (*N* = 6,145).

	CES-D		Binge		Painkiller		Hard Drugs		Suicide	
Demographics and IQ										
Age	.02	[−.01, .04]	.03*	[.00, .05]	.02	[−.00, .04]	.01	[−.01, .04]	.01	[−.01, .03]
Male	−.39***	[−.46, −.31]	−.36***	[−.44, −.29]	−.37***	[−.44, −0.30]	−.37***	[−.44, −.30]	−.37***	[−.44, −.30]
Hispanic	.24***	[.13, .36]	.24***	[.12, .36]	.24***	[.12, .36]	.24***	[.13, .36]	.24***	[.12, .36]
Black (non-Hispanic)	.41***	[.29, .53]	.38***	[.25, .50]	.39***	[.27, .51]	.40***	[.28, .52]	.41***	[.29, .53]
Asian (non-Hispanic)	.66***	[.48, .84]	.62***	[.44, .80]	.64***	[.46, .82]	.65***	[.46, .83]	.65***	[.46, .83]
Other (non-Hispanic)	.40	[−.03, .84]	.39	[−.04, .82]	.38	[−.05, .82]	.40	[−.03, .84]	.40	[−.03, .84]
Peabody test	.35***	[.30, .39]	.35***	[.30, .39]	.34***	[.30, .38]	.35***	[.30, .39]	.34***	[.30, .39]
Parents										
Occupation	.09***	[.06, .12]	.09***	[.06, .11]	.09***	[.06, .12]	.09***	[.06, .12]	.09***	[.06, .12]
Household income	.18***	[.15, .21]	.18***	[.15, .21]	.18***	[.15, .21]	.18***	[.15, .21]	.18***	[.15, .21]
Low educated parents	−.25***	[−.38, −.12]	−.27***	[−.40, −.13]	−.27***	[−.40, −.14]	−.26***	[−.40, −.13]	−.26***	[−.39, −.13]
Married parents	−.13*	[−.25, −.01]	−.14*	[−.26, −.02]	−.14*	[−.26, −.02]	−.12*	[−.24, −.01]	−.12*	[−.24, −.00]
Single parent at age 0	−.17***	[−.27, −.07]	−.17***	[−.27, −.07]	−.17***	[−.27, −.07]	−.17***	[−.27, −.07]	−.17***	[−.27, −.07]
Single parent at age 13	−.01	[−.12, .09]	−.02	[−.12, .09]	−.00	[−.11, .10]	−.01	[−.12, .09]	−.02	[−.12, .09]
Parent in jail	−.23***	[−.35, −.12]	−.23***	[−.34, −.11]	−.22***	[−.34, −.11]	−.23***	[−.35, −.12]	−.24***	[−.35, −.12]
Parent binges	−.07	[−.18, .04]	−.05	[−.16, .06]	−.07	[−.18, .05]	−.07	[−.18, .04]	−.07	[−.18, .04]
Parents talk about separation	−.15*	[−.27, −.03]	−.14*	[−.27, −.02]	−.14*	[−.26, −.01]	−.15*	[−.27, −.03]	−.15*	[−.27, −.03]
Parents fight at all	−.01	[−.11, .08]	−.01	[−.10, .09]	−.00	[−.10, .09]	−.01	[−.10, .09]	−.01	[−.11, .08]
Parents fight a lot	.08	[−.17, .33]	.07	[−.18, .33]	.06	[−.20, .31]	.06	[−.19, .32]	.07	[−.18, .32]
Neighborhood										
Low proportion White	−.12*	[−.23, −.01]	−.13*	[−.24, −.02]	−.12*	[−.23, −.01]	−.13*	[−.24, −.01]	−.12*	[−.23, −.01]
Low median household income	.04	[−.06, .15]	.04	[−.06, .15]	.04	[−.07, .14]	−.05	[−.06, .15]	.04	[−.06, .15]
High unemployment	.01	[−.09, .11]	.01	[−.10, .11]	.01	[−.10, .11]	.01	[−.09, .11]	.01	[−.09, .11]
School										
Large class size	.02	[−.08, .11]	.01	[−.09, .10]	.01	[−.08, .11]	.02	[−.07, .11]	.02	[−.07, .11]
Low ratio of teachers with master’s	−.03	[−.13, .07]	−.04	[−.14, .06]	−.04	[−.14, .06]	−.03	[−.13, .07]	−.03	[−.13, .07]
High proportion of students held back	.20***	[.10, .30]	.19***	[.09, .30]	.19***	[.09, .30]	.20***	[.10, .30]	.20***	[.10, .30]
High dropout rate	−.23***	[−.32, −.14]	−.23***	[−.32, −.14]	−.23***	[−.31, −.14]	−.23***	[−.32, −.14]	−.23***	[−.32, −.15]
Low attendance	−.15*	[−.26, −.03]	−.14*	[−.26, −.02]	−.14*	[−.26, −.02]	−.15*	[−.27, −.03]	−.15*	[−.27, −.03]
Childhood adversities										
Run away from home	−.26***	[−.40, −.12]	−.27***	[−.42, −.13]	−.25***	[−.39, −.10]	−.27***	[−.42, −.13]	−.29***	[−.43, −.14]
Knife pulled at respondent	−.43***	[−.55, −.30]	−.41***	[−.53, −.28]	−.38***	[−.51, −.26]	−.43***	[−.55, −.31]	−.44***	[−.56, −.31]
Parents don’t care about respondent	−.04	[−.25, .17]	−.09	[−.30, .12]	−.07	[−.28, .15]	−.08	[−.29, .13]	−.10	[−.31, .12]
Household member touched in sexual way	−.01	[−.17, .16]	−.01	[−.18, .16]	.00	[−.17, .17]	−.01	[−.18, .15]	−.01	[−.17, .16]
Household member kicked/hit/thrown	−.08	[−.17, .02]	−.07	[−.17, .02]	−.07	[−.17, .02]	−.08	[−.17, .02]	−.08	[−.17, .02]
Household received welfare during childhood	−.56***	[−.66, −.47]	−.57***	[−.66, −.47]	−.56***	[−.65, −.46]	−.57***	[−.66, −.47]	−.57***	[−.66, −.48]
No health insurance	−.14*	[−.27, −.02]	−.14*	[−.26, −.01]	−.15*	[−.28, −.02]	−.14*	[−.27, −.01]	−.13*	[−.26, −.00]
Interview observations										
Evidence of drinking	−.10	[−.30, .11]	−.09	[−.29, .11]	−.09	[−.29, .11]	−.10	[−.30, .10]	−.10	[−.30, .10]
Safety concerns	−.02	[−.21, .17]	−.04	[−.23, .16]	−.02	[−.21, .17]	−.03	[−.22, .16]	−.04	[−.23, .16]
Poor dwelling condition	−.17**	[−.29, −.05]	−.17**	[−.30, −.05]	−.17**	[−.30, −.05]	−.18**	[−.30, −.05]	−.17**	[−.30, −.05]
Poor grooming	−.09	[−.32, .14]	−.08	[−.31, .15]	−.06	[−.29, .17]	−.09	[−.32, .15]	−.12	[−.35, .11]
Health selection										
Wave I equivalent measure	−.03**	[−.04, −.01]	−.23***	[−.32, −.14]	−.31***	[−.42, −.20]	−.22*	[−.40, −.03]	−.03	[−.13, .08]
Intercept	−.73***	[−1.09, −.38]	−.88***	[−1.24, −.52]	−.79***	[−1.15, −.43]	−.74***	[−1.10, −.39]	−.71***	[−1.07, −.36]
Akaike information criterion	6,962.71		6,923.75		6,914.62		6,977.40		6,968.58	
Bayesian information criterion	7,225.26		7,186.10		7,176.83		7,239.98		7,231.06	
Pseudo *R*^2^	.189		.189		.188		.188		.187	
Observations	6,145		6,145		6,145		6,145		6,145	

*Source*: National Longitudinal Study of Adolescent to Adult Health.

*Note*: The 95% confidence intervals are in brackets. CES-D = Center for Epidemiologic Studies Depression Scale.

* *p* < .05, **p < .01, ***p < .001.

**Figure 1.**
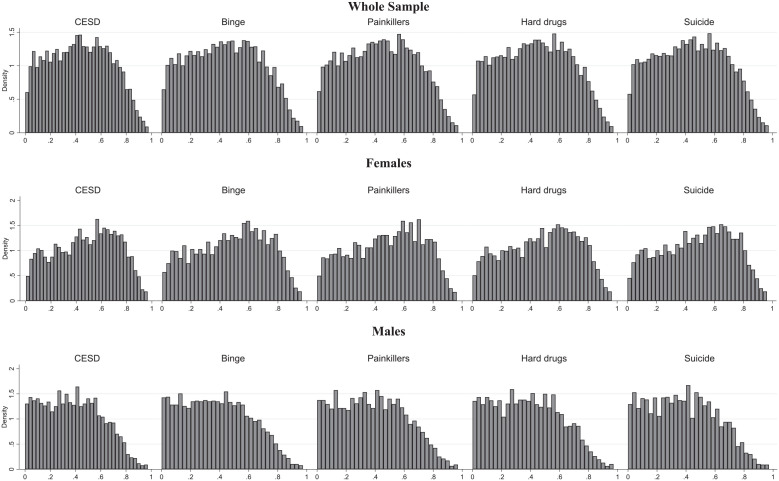
Propensity Score Histograms, Models with the Full Set of Variables, including Initial Values of Outcome Measures. *Source*: National Longitudinal Study of Adolescent to Adult Health. *Note*: Propensity scores were estimated by a probit regression model of college completion on the set of precollege covariates. The number of observations (N = 6,145) is the same as in Table 1 and Tables S2 through S6 in the online version of the article. CES-D = Center for Epidemiologic Studies Depression Scale.

Figure 1 shows the distribution of the derived propensity scores in the whole sample and by gender. These propensity scores are shown specifically for all DoD-related outcomes, and the observed differences between them stem from accounting for the initial values of these outcomes. The depicted propensity scores resemble normal distribution but are slightly right-skewed, suggesting that fewer individuals have a high likelihood of college completion than individuals with a low likelihood of college completion. This pattern in the overall sample primarily derives from male Add Health participants whose likelihood of college completion sharply declines after a .6 propensity score. By contrast, the decline mainly occurs for females after a .8 propensity score.

### Main HTE Estimates

**Figure 2.**
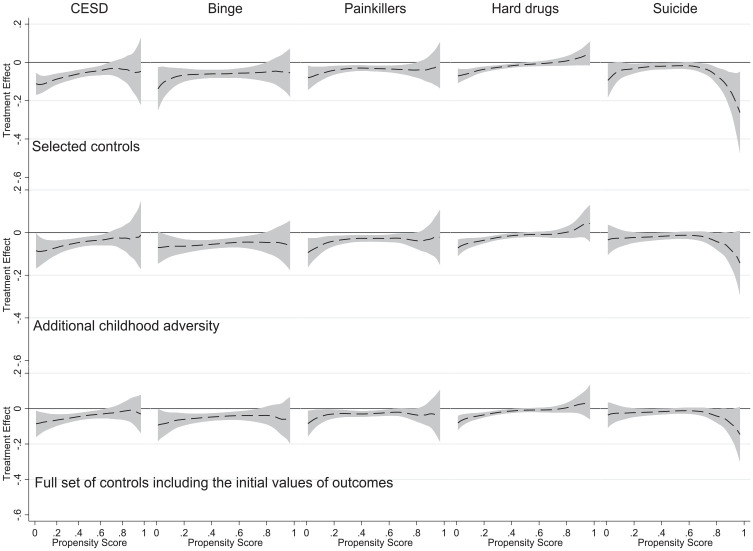
Heterogeneous Treatment Effect Analysis Using the Smoothing-Differencing Method. *Source*: National Longitudinal Study of Adolescent to Adult Health. *Note*: The gray area indicates 95% confidence bands. Propensity scores were estimated by a probit regression model of college completion on the set of precollege covariates. The number of observations (N = 6,145) is the same as in Table 1. CES-D = Center for Epidemiologic Studies Depression Scale.

Table 2 presents descriptive statistics for DoD outcomes and differences between the samples of individuals with and without college completion. It is clear that having a college degree is linked to a much lower likelihood of adverse DoD-related outcomes. Yet this association does not account for the selection mechanism affecting college completion. Based on the generated propensity scores, we present HTE analyses for five DoD outcomes of interest measured at Add Health Wave V. Figure 2 shows the DoD-related outcomes for those individuals who have attained college degrees compared to those who did not. In addition, these estimates are shown across the generated spectrum of propensity scores. We present the main results in three steps: First, we only account for the variables described in the treatment selection variables section; second, we introduce additional childhood adversity measures; and third, we further control for the health selection mechanism by including in the models the initial values of the respective outcomes related to DoD.

**Table 2.** Descriptive Statistics of Deaths of Despair Outcomes by Completed College Education.

			Total Sample		Sample without Completed College		Sample with Completed College	
Variable	Minimum	Maximum	Mean	*SD*	Mean	*SD*	Mean	*SD*
CES-D	.00	1.00	.15	.36	.18	.39	.11***	.31
Binge drinking	.00	1.00	.15	.35	.18	.38	.10***	.30
Painkiller misuse	.00	1.00	.07	.25	.09	.28	.04***	.20
Hard drugs consumption	.00	1.00	.03	.18	.04	.19	.02***	.15
Suicidal thoughts	.00	1.00	.07	.25	.08	.26	.05***	.22

*Source*: National Longitudinal Study of Adolescent to Adult Health.

*Note*: Test for the difference between means of samples with and without completed college education. *N* observations = 6,145. CES-D = Center for Epidemiologic Studies Depression Scale.

*** *p* < .001.

The derived results are consistent across DoD-related outcomes and suggest that completing college reduces the likelihood of depressive symptoms, binge drinking, abusing prescription drugs, and consuming hard drugs, and this effect is more salient among individuals who have a low propensity for graduating from college but are still able to do it. The only exception is suicidal thoughts, which appears to be lower among those with degrees and the highest propensity for college completion in the top row of Figure 2. Yet this association becomes insignificant when the additional childhood adversity measures and health selection mechanisms are accounted for in the second and third rows of Figure 2. We can also see that the introduction of a more comprehensive set of predictors of college completion and the initial values of DoD-related outcomes do not change the overall finding that individuals with a lower propensity of college completion have better outcomes related to depressive symptoms, binge drinking, abusing prescription drugs, and consuming hard drugs.

If we consider specific outcomes related to DoD, Figure 2 shows that college completion positively affects mental health: CES-D is noticeably lower for individuals with degrees in the low to middle propensity score range (.0–.6). Above this range, the effect of completing college on CES-D is not significantly different from 0 at the 95% confidence level. Figure 2 also indicates that the significant effect of a college degree on binge drinking and painkiller use takes place within the .0 to .8 range of propensity score. By contrast, for those who are most likely to finish college, the impact appears to be insignificant. For the hard drugs consumption component of DoD-related outcomes, we see that the propensity score range of the significant effect of college completion is narrower (between .0 and .4). Yet this association is steeper with noticeably smaller confidence intervals when compared with other outcomes. Furthermore, the shape of the graph provides some indication that a college degree among the most advantaged individuals could be related to a higher consumption of hard drugs. Still, these estimates’ confidence intervals are large, overlapping with the zero reference line.

### Gender Differences in HTE Estimates

**Figure 3.**
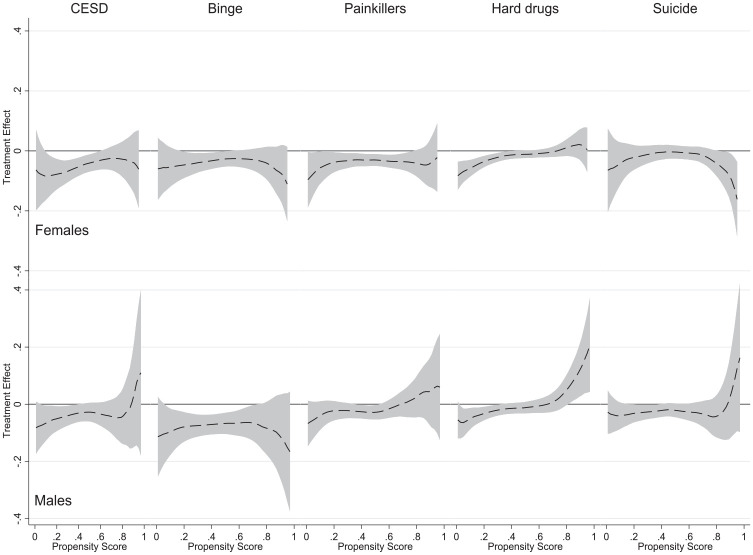
Heterogeneous Treatment Effect Analysis Using the Smoothing-Differencing Method. *Source*: National Longitudinal Study of Adolescent to Adult Health. *Note*: The gray area indicates 95% confidence bands. Propensity scores were estimated by a probit regression model of college completion on the set of precollege covariates. The number of observations is as shown in Tables S2 and S3 in the online version of the article. CES-D = Center for Epidemiologic Studies Depression Scale.

To estimate HTEs separately for females and males, we first generate gender-specific propensity scores for college completion (as shown in Tables S2 and S3 in the online version of the article). The HTE estimates by gender are shown in Figure 3. They are based on identical specifications to the estimates in the bottom row of Figure 2 and suggest that there are some differences between males and females. For the mental health of females, measured with CES-D, in the low range of propensity score, between .1 and .6, the positive impact of graduating from college is statistically significant. The results differ for males, where the impact is only observed for propensity scores between .1 and .4. HTEs concerning binge drinking are more pronounced in the case of males. The opposite can be observed for the use of painkillers and hard drugs, for which females have a wider range of propensity scores where the effect of college completion is significant. Figure 3 also reveals that females who graduated from college think less about committing suicide, but only those most likely to have college degrees (propensity scores above .8). The estimated curve has an opposite shape for males, but the confidence intervals consistently overlap with the zero reference line.

### Racial-Ethnic Differences

**Figure 4.**
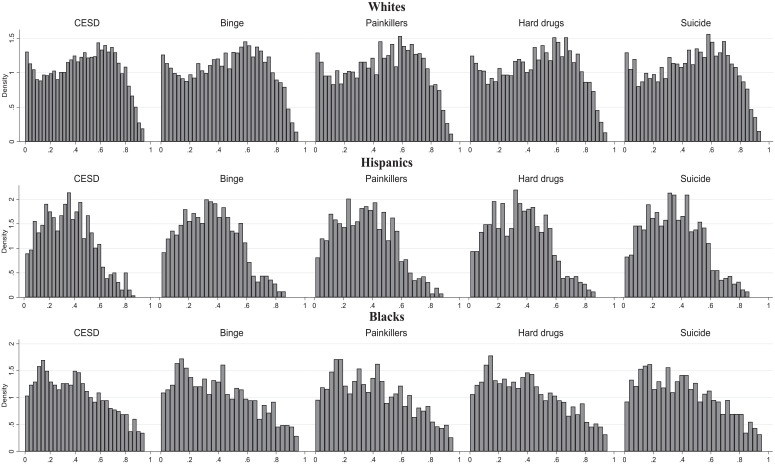
Propensity Score Histograms, Models with the Full Set of Variables, Including Initial Values of Outcome Measures. *Source*: National Longitudinal Study of Adolescent to Adult Health. *Note*: Propensity scores were estimated by a probit regression model of college completion on the set of precollege covariates. The number of observations is as in Table 1 (N = 6,145) Tables S4 to S6 in the online version of the article. CES-D = Center for Epidemiologic Studies Depression Scale.

Figure 4 presents the generated propensity scores from models where the sample is split by race-ethnicity (see also Tables S4–S6 in the online version of the article). The presented distribution suggests that the propensity scores vary across racial-ethnic groups, with Hispanic respondents having much lower propensity score densities above .6. The propensity score distribution for Black respondents is more equal, but compared to White respondents, still lower densities can be observed above .8. These structural racial-ethnic inequalities are likely to affect HTE estimates, which we describe in the following.

**Figure 5.**
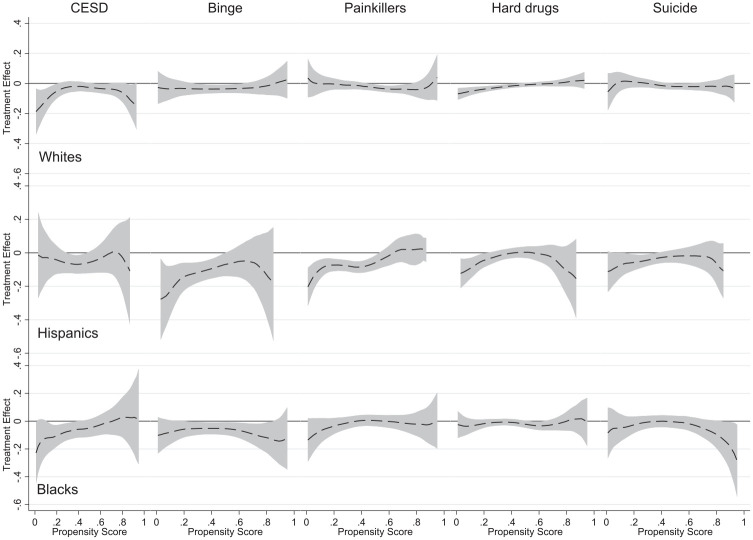
Heterogeneous Treatment Effect Analysis Using the Smoothing-Differencing Method. *Source*: National Longitudinal Study of Adolescent to Adult Health. *Note*: The gray area indicates 95% confidence bands. Propensity scores were estimated by a probit regression model of college completion on the set of precollege covariates. The number of observations is as in Tables S4 to S6 in the online version of the article.

Further analyses of the sample split with respect to race-ethnicity in Figure 5 reveal additional differences in this demographic characteristic. The HTE estimates for depressive symptoms, binge drinking, and misuse of painkillers in the case of White respondents are different compared to the results for Hispanic and Black respondents. For the considered racial-ethnic groups, the positive effect of college completion on mental health is observed at slightly different propensity scores—for White respondents at the low range, for Hispanic respondents at the middle range, and for Black respondents at the low-middle range. For binge drinking and painkiller consumption, the effect of college completion is significant in the middle and middle-upper range of propensity scores for White respondents and the low and middle range for Hispanic respondents. By contrast, for Black respondents, college completion is linked with less binge drinking in the upper-middle range of propensity score. For hard drug consumption, both White and Hispanic respondents benefit from college completion at the lower end of the propensity score. Figure 5 also indicates that, respectively, at the middle and low ranges, college education among the latter racial-ethnic groups is associated with a lower likelihood of having suicidal thoughts.

### Robustness Checks with Alternative Model and Treatment Specifications

To check the robustness of our findings with alternative model specifications, in the supplementary materials, we present homogeneous effects of college completion for the five considered outcomes related to DoD (Xie et al. 2012). First, as shown in Table S7 in the online version of the article, we derive results with only demographic controls such as age, gender, race-ethnicity, and the binary college attainment variable without calculating propensity scores. The derived coefficients for the college completion variable seem slightly larger but in line with what we observed in the main analyses shown in Figure 2. Next, as shown in Table S8 in the online version of the article, we rerun the regression with only the college degree variable and the generated propensity score, and as shown in Table S9 in the online version of the article, we enter the propensity score in the form of deciles. The estimated effect sizes for the college degree variable are slightly smaller and seem to closely match the average effect of a college degree for the considered DoD-related outcomes shown in Figure 2. We also tested the robustness of the findings with the stratification-multilevel HTE method (see Figure S1 in the online version of the article; Brand and Thomas 2013). These results do not significantly differ from our main estimates in Figure 2.

We also consider a broader and narrower treatment variable. Specifically, based on the same attainment measure, a binary indicator is constructed. It is equal to 1 if the respondent completed at least some college. The narrower measure takes the value of 1 if only postgraduate studies were completed. As shown in Figure S2 in the online version of the article, slight differences can be observed in the case of completed college and some college treatment variables’ impact on painkiller use. The effect is different from zero for propensity .5 to .6. Similarly, for hard drugs, the positive effect of completing some college can be observed in a narrower propensity range (.2–.4). In the case of completing postgraduate studies, the treatment effect is less pronounced for CES-D. Furthermore, it appears that after the narrowing down of the treatment measure, the attainment effect has shifted toward higher ranges of the propensity score for hard drug use. In the next step, we tested if changing the default methods of using logit instead of probit function to estimate the propensity score affects the main estimates. The results presented in Figure S3 in the online version of the article align with the main results shown in Figure 2.

We also examine if our main findings hold if college completion eight years earlier at Wave 4 is used instead of Wave 5 attainment. The aim here is to address a potential concern that in some cases, the outcomes may occur before the treatment takes place with the original approach, even though based on attainment before Wave 5 of the survey, most individuals have already completed educational trajectories (Bernardi and Ballarino 2016). Figure S4 in the online version of the article presents results in line with our previous estimates. Only in the case of binge drinking does the downward slope in the higher ranges of the propensity score appear to be steeper.

As part of robustness checks, we also use an alternative approach to estimate propensity scores by implementing the Itpscore, as discussed in the data and methods section. This method allowed us to narrow down the number of variables used in the modeling step by approximately half and resulted in the inclusion of higher-order and interaction terms. The results presented in Figure S5 in the online version of the article confirm our main findings. The most noticeable difference can be observed in the case of painkiller use at low values of the propensity score. Changing the improvement threshold that must be satisfied for the inclusion of linear and interaction terms does affect the number of variables used; however, the HTE estimates remain largely the same as in Figure S6 in the online version of the article.

Finally, to understand the source of the differences in the estimated effects, in Table S10 in the online version of the article, we present the main estimates by college completion and the propensity of college completion. The table presents means for the five outcomes by college completion and propensity strata. The results indicate again that DoD outcomes are linked to college completion to a greater extent at the lower levels of propensity score, particularly for CES-D, painkillers, and suicide thoughts. This suggests that the stronger negative effect of college on suicide may be attributed to the high upper tail of the propensity distribution.

## Discussion

Educational attainment, particularly having a college degree, is one of the most important and extensively researched social determinants of physical and psychological well-being (Raphael 2016). High socioeconomic disparity in college completion might be one of the reasons for significant health inequalities in the United States (Jackson and Holzman 2020). Education also features prominently in the emerging research on the explanations of DoD. The growing share of deaths from substance use disorders and self-harm has been referred to as a socially patterned epidemic and the American society’s signal characteristic in the twenty-first century (King et al. 2022). The evidence suggests that the prevalence of DoD is lower among college-educated individuals than in the rest of the population, but college completion does not eliminate the likelihood of these types of deaths (Case and Deaton 2022). Yet it is largely unknown if the likelihood of college completion influences the beneficial effects of education for DoD-related outcomes.

To address the main research question of this study, using panel data from the Add Health survey, we calculated propensity scores for individuals’ likelihood of college completion based on a wide array of childhood and adolescent characteristics predicting later life college completion. After each participant was assigned propensity scores varying from just above 0 (very unlikely to complete college) to just below 1 (very likely to complete college), we tested the effect of college completion on five selected outcome measures that come close to DoD—depressive symptoms, binge drinking, misuse of painkillers, hard drug consumption, and suicidal thoughts—along the full range of the propensity scores. Our goal was to explore if there were systemic differences in the effect of college completion on outcomes related to DoD conditioned by the likelihood of college completion. To achieve this goal, we employed a recently developed HTE analytical tool using the SD approach (Trinidad 2021).

In line with our expectation, we found that the most disadvantaged individuals in the Add Health survey were the ones for whom a college degree was linked with a lower likelihood of having depressive symptoms, binge drinking, misusing painkillers, and consuming hard drugs in comparison to those with low propensity score and no college degree. These effects are statistically significant even after accounting for health selection effects by including Wave I measures of the corresponding DoD-related outcomes in the estimation of the propensity scores. The only considered measure for which, in some models, we find the effect of college completion in the higher ranges of the propensity score is suicidal thoughts. The results provide some indication that the negative effect of college completion on considering suicide is stronger among those who have the highest likelihood of college completion. One possible explanation of this finding is that there is, expectedly, a strong educational gradient in suicide in the United States. Those with a college degree have much lower rates of suicide. More important for the interpretation of our finding is that among college-educated individuals, the primary reasons for suicide are job-related stressors (Phillips and Hempstead 2017), and arguably, the more advantaged individuals with material and psychosocial resources and a higher likelihood of college completion are better equipped to cope with these problems.

Our findings align with the “rising from the rags,” “resource substitution,” and reference group theoretical perspectives. After experiencing intergenerational upward mobility, disadvantaged individuals seem to benefit from their college degrees more than those who did not have to overcome the significant obstacles in their educational trajectories. In other words, the effect of college completion on DoD-related outcomes is greater for those with fewer alternative resources, such as family support, parental wealth, or beneficial social connections. College education not only can positively affect the sense of personal control and mastery but also can help individuals acquire other types of resources that they would not otherwise possess, such as higher earnings, more stable employment, or better quality housing (Settels 2022). Future research should explore specific channels through which college education among disadvantaged individuals improves their DoD-related outcomes.

We also explored the gender and racial-ethnic differences in HTEs of college completion across five DoD-related measures. We found varying results but no systematic differences between the considered gender and racial-ethnic groups. For some outcomes, such as depressive symptoms and misuse of painkillers, females benefit more from college completion, whereas for other outcomes, such as binge drinking and hard drug consumption, the gradient of college completion by propensity score is more visible for males. In terms of thinking less about committing suicide, graduating from college brings benefits to females in the higher propensity score range. Furthermore, for racial-ethnic differences, treatment effects across the propensity score range are more salient for White (e.g., CES-D, hard drugs) and Hispanic individuals (e.g., binge drinking, painkillers) than for Black individuals. Yet this can be potentially explained by the differences in sample sizes by race-ethnicity and by generally lower chances of Black individuals completing college due to, among other factors, systemic racism in the United States (Barber et al. 2020).

Our study has its limitations. First, because Add Health participants are relatively young and the number of deaths in general and DoD in particular is very low, we could not analyze the actual mortality outcomes of individuals from the considered causes, but rather, we investigated the outcomes that are related to DoD. It remains to be tested how the HTEs of college education are reflected in actual mortality associated with DoD. Second, we used the cutting-edge statistical approach to generate propensity scores for college completion of Add Health participants and then estimate HTEs. Yet we cannot assert that the associations that we found are causal. It is possible that individuals’ unobserved characteristics, such as genetic predisposition and personality traits through some form of selection, might affect both college completion and DoD-related outcomes. Third, despite using the high-quality panel survey, the data used in our study did not allow us to comprehensively investigate HTEs of college completion for less numerous racial-ethnic groups or observe changes across time along with increasing income and educational inequalities in the United States (Bloome, Dyer, and Zhou 2018).

## Conclusion

Our results indicate that college completion has heterogeneous effects on DoD-related outcomes in the United States, with individuals less likely to complete college deriving more significant benefits. This underscores the importance of targeted educational policies, such as free community college and financial aid, to support high-risk students effectively. By focusing resources on these individuals, policymakers can reduce inequality, enhance social mobility, and lower societal costs associated with substance abuse and mental health issues. Although our study found varying results by gender and racial-ethnic groups, no systematic differences emerged. Future research should investigate the mechanisms behind these heterogeneous effects and examine whether these associations are causal to better inform policy development.

## Supplemental Material

sj-docx-1-hsb-10.1177_00221465241291845 – Supplemental material for The Heterogeneous Effects of College Education on Outcomes Related to Deaths of Despair Supplemental material, sj-docx-1-hsb-10.1177_00221465241291845 for The Heterogeneous Effects of College Education on Outcomes Related to Deaths of Despair by Grzegorz Bulczak, Alexi Gugushvili and Jonathan Koltai in Journal of Health and Social Behavior

## Notes

1. The access to the restricted data underlying this article is provided by the National Longitudinal Study of Adolescent to Adult Health, Carolina Population Center, University of North Carolina at Chapel Hill at https://addhealth.cpc.unc.edu/. The Stata code for the presented analysis is available via the Open Science Framework (Gugushvili 2024).
