# Supplementary material for: The Heterogeneous Effects of College Education on Outcomes Related to Deaths of Despair
Source: J Health Soc Behav. 2024 Nov 14;66(3):357–78. doi: 10.1177/00221465241291845 (PMC12394775; doi:10.1177/00221465241291845)
Supplement: sj-docx-1-hsb-10.1177_00221465241291845 – Supplemental material for The Heterogeneous Effects of College Education on Outcomes Related to Deaths of Despair [file sj-docx-1-hsb-10.1177_00221465241291845.docx]

**Journal** of **Health**

and **Social Behavior**

OFFICIAL JOURNAL OF THE AMERICAN SOCIOLOGICAL ASSOCIATION

**ONLINE SUPPLEMENT**

**to article in**

Journal of Health and Social Behavior

**The Heterogeneous Effects of College Education on Outcomes Related to Deaths of Despair**

**Grzegorz Bulczak**

*Gdynia Maritime University*

**Alexi Gugushvili**

*University of Oslo*

**Jonathan Koltai**

*Independent researcher*

Figure S1. Heterogeneous Treatment Effect Analysis Using the Stratification-Multilevel Method

Note: Bars indicate 95% confidence bands. Propensity scores were estimated by a probit regression model of college completion on the set of precollege covariates. Number of observations as in Table 1. *Source:* Add Health.

Figure S2. Heterogeneous Treatment Effect Analysis Using the Smoothing-Differencing Method, Narrower and Broader Treatment Variables

Note: Grey area indicates 95% confidence bands. Propensity scores were estimated by a probit regression model of college completion on the set of precollege covariates. Number of observations as in Table 1. *Source:* Add Health.

Figure S3. Heterogeneous Treatment Effect Analysis Using the Smoothing-Differencing Method-Logit Model

Note: Grey area indicates 95% confidence bands. Propensity scores were estimated by a logit regression model of college completion on the set of precollege covariates. Number of observations as in Table 1. *Source:* Add Health.

Figure S4. Heterogeneous Treatment Effect Analysis Using the Smoothing-Differencing Method-Probit Model With Wave 4 College Completion

Note: Grey area indicates 95% confidence bands. Propensity scores were estimated by a logit regression model of college completion on the set of precollege covariates. Number of observations as in Table 1. *Source:* Add Health.

Figure S5. Heterogeneous Treatment Effect Analysis Using the Smoothing-Differencing Method- Itpscore Logistic Model

Note: Grey area indicates 95% confidence bands. Number of observations as in Table 1.Propensity scores were estimated by a logit regression model of college completion on a narrowed set of precollege covariates: HH income, HH received welfare during childhood, Pbody test, High proportion of students held back, Low median HH income, High dropout rate, Parent in jail, Parental occupation, Gender, Pbody test^2. *Source:* Add Health.

Figure S6. Heterogeneous Treatment Effect Analysis Using the Smoothing-Differencing Method- Itpscore Logistic Model with a Higher Improvement Threshold

Note: Grey area indicates 95% confidence bands. Number of observations as in Table 1. Propensity scores were estimated by a logit regression model of college completion on a broader set of precollege covariates: HH income, HH received welfare during childhood, Pbody test, High proportion of students held back, Low median HH income, High dropout rate, Parent in jail, Parental occupation, Gender, Parents fight at all, Knife pulled at resp., No health insurance, HH member kicked/hit/thrown, Single parent at age 0 and 13, Evidence of drinking Black(nonHisp), Asian (nonHisp), Hispanic Low ratio of teachers with masters, CESD wave 1, Parent binges, Parental education, Poor dwelling condition, Run away from home, Parents talk about separation, Married parents, HH member touched in sexual way, High unemployment neighborhood; Interactions: HH income#Hispanic, HH income#Black(nonHisp), Parental occupation#Parental occupation, Male#dwelling condition. *Source:* Add Health.

Table S1: Descriptive Statistics

|  | Variable | Mean | SD | Min | Max |
| --- | --- | --- | --- | --- | --- |
| Treatment | Completed college wave 5 | 0.41 | 0.49 | 0.00 | 1.00 |
|  | Completed some college wave 5 | 0.53 | 0.50 | 0.00 | 1.00 |
|  | Completed postgraduate studies wave 5 | 0.16 | 0.37 | 0.00 | 1.00 |
|  | Completed college wave 4 | 0.32 | 0.46 | 0.00 | 1.00 |
| Demographics and IQ | Age | 15.15 | 1.74 | 11.00 | 20.00 |
|  | Male | 0.50 | 0.50 | 0.00 | 1.00 |
|  | Hispanic | 0.17 | 0.38 | 0.00 | 1.00 |
|  | Black (non-Hispanic) | 0.22 | 0.42 | 0.00 | 1.00 |
|  | White (non-Hispanic) | 0.52 | 0.50 | 0.00 | 1.00 |
|  | Asian (non-Hispanic | 0.07 | 0.25 | 0.00 | 1.00 |
|  | Other(non-Hispanic | 0.01 | 0.09 | 0.00 | 1.00 |
|  | Pbody test | 0.00 | 1.00 | -5.71 | 3.06 |
| Parents | Occupation | 3.00 | 1.36 | 1.00 | 5.00 |
|  | HH income | 2.96 | 1.43 | 1.00 | 5.00 |
|  | Parents with low education | 0.16 | 0.36 | 0.00 | 1.00 |
|  | Married parents | 0.70 | 0.46 | 0.00 | 1.00 |
|  | Single parent at age=0 | 0.23 | 0.42 | 0.00 | 1.00 |
|  | Single parent at age=13 | 0.28 | 0.45 | 0.00 | 1.00 |
|  | Parents talked about separation | 0.11 | 0.31 | 0.00 | 1.00 |
|  | Parent in jail during childhood | 0.13 | 0.34 | 0.00 | 1.00 |
|  | Parent binged | 0.13 | 0.33 | 0.00 | 1.00 |
|  | Parents fight at all | 0.62 | 0.48 | 0.00 | 1.00 |
|  | Parents fight a lot | 0.02 | 0.16 | 0.00 | 1.00 |
| Neighborhood W1 | Proportion white | 0.36 | 0.48 | 0.00 | 1.00 |
|  | Low median HH income | 0.22 | 0.42 | 0.00 | 1.00 |
|  | High unemployment | 0.23 | 0.42 | 0.00 | 1.00 |
| School W1 | Class size is 30 or more | 0.27 | 0.44 | 0.00 | 1.00 |
|  | Low proportion of teacher with maters | 0.20 | 0.40 | 0.00 | 1.00 |
|  | High proportion of students held back | 0.22 | 0.41 | 0.00 | 1.00 |
|  | High dropout rate | 0.32 | 0.47 | 0.00 | 1.00 |
|  | Low attendance | 0.24 | 0.43 | 0.00 | 1.00 |
| Childhood adversities | Run away from home | 0.09 | 0.28 | 0.00 | 1.00 |
|  | Knife pulled at resp. | 0.13 | 0.34 | 0.00 | 1.00 |
|  | Parents don’t care about resp. | 0.04 | 0.21 | 0.00 | 1.00 |
|  | HH member touched in sexual way | 0.05 | 0.22 | 0.00 | 1.00 |
|  | HH member kicked/hit/thrown | 0.19 | 0.39 | 0.00 | 1.00 |
|  | HH received welfare during childhood | 0.24 | 0.43 | 0.00 | 1.00 |
|  | No health insurance | 0.13 | 0.33 | 0.00 | 1.00 |
| Interviewers’ observations W1 | Evidence of drinking | 0.04 | 0.19 | 0.00 | 1.00 |
|  | Concerns for safety | 0.05 | 0.21 | 0.00 | 1.00 |
|  | Poor dwelling condition | 0.15 | 0.36 | 0.00 | 1.00 |
|  | Poor grooming of resp. | 0.04 | 0.20 | 0.00 | 1.00 |
| Outcomes at W1 | CESD | 2.44 | 2.29 | 0.00 | 12.00 |
|  | Binge drinking | 0.22 | 0.41 | 0.00 | 1.00 |
|  | Painkiller misuse | 0.16 | 0.36 | 0.00 | 1.00 |
|  | Hard drugs consumption | 0.04 | 0.20 | 0.00 | 1.00 |
|  | Suicidal thoughts | 0.13 | 0.34 | 0.00 | 1.00 |

*Source:* Add Health.

Table S2: Probit Regression Estimates Predicting College Completion, Females Only

|  | CESD |  | Binge |  | Painkiller |  | Hard drugs |  | Suicide |  |
| --- | --- | --- | --- | --- | --- | --- | --- | --- | --- | --- |
| *Demographics and IQ* |  |  |  |  |  |  |  |  |  |  |
| Age | 0.03^*^ | [0.00,0.06] | 0.04^*^ | [0.01,0.07] | 0.03^*^ | [0.00,0.06] | 0.03 | [-0.00,0.06] | 0.02 | [-0.00,0.05] |
| Hispanic | 0.20^*^ | [0.04,0.35] | 0.19^*^ | [0.03,0.34] | 0.19^*^ | [0.04,0.34] | 0.20^*^ | [0.04,0.35] | 0.19^*^ | [0.03,0.34] |
| Black(nonHisp) | 0.50^***^ | [0.34,0.66] | 0.48^***^ | [0.32,0.64] | 0.48^***^ | [0.32,0.64] | 0.49^***^ | [0.33,0.65] | 0.50^***^ | [0.34,0.66] |
| Asian(nonHisp) | 0.49^***^ | [0.24,0.75] | 0.45^***^ | [0.19,0.71] | 0.46^***^ | [0.20,0.71] | 0.48^***^ | [0.22,0.74] | 0.48^***^ | [0.23,0.74] |
| Other(nonHisp) | -0.24 | [-0.89,0.40] | -0.25 | [-0.89,0.40] | -0.24 | [-0.89,0.41] | -0.22 | [-0.87,0.42] | -0.23 | [-0.87,0.41] |
| Pbody test | 0.35^***^ | [0.29,0.41] | 0.34^***^ | [0.29,0.40] | 0.34^***^ | [0.28,0.40] | 0.34^***^ | [0.29,0.40] | 0.34^***^ | [0.29,0.40] |
| *Parents* |  |  |  |  |  |  |  |  |  |  |
| Occupation | 0.10^***^ | [0.06,0.14] | 0.10^***^ | [0.06,0.14] | 0.10^***^ | [0.06,0.14] | 0.10^***^ | [0.06,0.14] | 0.10^***^ | [0.07,0.14] |
| HH income | 0.15^***^ | [0.10,0.19] | 0.14^***^ | [0.10,0.19] | 0.15^***^ | [0.10,0.19] | 0.14^***^ | [0.10,0.18] | 0.14^***^ | [0.10,0.18] |
| Low edu. parents | -0.28^***^ | [-0.45,-0.12] | -0.31^***^ | [-0.48,-0.15] | -0.32^***^ | [-0.48,-0.15] | -0.30^***^ | [-0.47,-0.14] | -0.29^***^ | [-0.46,-0.13] |
| Married parents | -0.08 | [-0.24,0.07] | -0.09 | [-0.24,0.07] | -0.09 | [-0.24,0.07] | -0.07 | [-0.23,0.08] | -0.07 | [-0.22,0.08] |
| Single parent at age=0 | -0.16^*^ | [-0.28,-0.03] | -0.16^*^ | [-0.29,-0.03] | -0.17^*^ | [-0.30,-0.04] | -0.16^*^ | [-0.29,-0.03] | -0.16^*^ | [-0.28,-0.03] |
| Single parent at age=13 | 0.06 | [-0.07,0.20] | 0.06 | [-0.08,0.20] | 0.08 | [-0.06,0.22] | 0.07 | [-0.07,0.20] | 0.06 | [-0.08,0.20] |
| Parent in jail | -0.16^*^ | [-0.31,-0.02] | -0.16^*^ | [-0.31,-0.02] | -0.15^*^ | [-0.30,-0.00] | -0.17^*^ | [-0.31,-0.02] | -0.17^*^ | [-0.32,-0.03] |
| Parent binges | -0.07 | [-0.22,0.07] | -0.06 | [-0.20,0.09] | -0.07 | [-0.21,0.07] | -0.08 | [-0.22,0.07] | -0.07 | [-0.22,0.07] |
| Parents talk about separation | -0.09 | [-0.25,0.07] | -0.08 | [-0.24,0.08] | -0.07 | [-0.23,0.09] | -0.09 | [-0.25,0.07] | -0.09 | [-0.25,0.07] |
| Parents fight at all | 0.05 | [-0.08,0.17] | 0.05 | [-0.08,0.18] | 0.05 | [-0.07,0.18] | 0.05 | [-0.07,0.18] | 0.05 | [-0.08,0.17] |
| Parents fight a lot | -0.15 | [-0.48,0.18] | -0.17 | [-0.50,0.16] | -0.17 | [-0.50,0.16] | -0.17 | [-0.50,0.16] | -0.16 | [-0.49,0.17] |
| *Neighborhood* |  |  |  |  |  |  |  |  |  |  |
| Low proportion White | -0.13 | [-0.27,0.02] | -0.13 | [-0.28,0.02] | -0.13 | [-0.28,0.02] | -0.13 | [-0.28,0.01] | -0.12 | [-0.27,0.02] |
| Low median HH income | 0.01 | [-0.13,0.15] | 0.00 | [-0.13,0.14] | -0.01 | [-0.14,0.13] | 0.01 | [-0.13,0.14] | 0.00 | [-0.14,0.14] |
| High unemployment | -0.02 | [-0.15,0.11] | -0.02 | [-0.15,0.11] | -0.02 | [-0.16,0.11] | -0.02 | [-0.15,0.11] | -0.02 | [-0.15,0.11] |
| *School* |  |  |  |  |  |  |  |  |  |  |
| Large class size | 0.01 | [-0.11,0.13] | -0.01 | [-0.14,0.11] | 0.00 | [-0.12,0.13] | 0.01 | [-0.11,0.14] | 0.00 | [-0.12,0.13] |
| Low ratio of teachers with masters | -0.03 | [-0.16,0.10] | -0.04 | [-0.17,0.09] | -0.04 | [-0.17,0.09] | -0.04 | [-0.16,0.09] | -0.04 | [-0.17,0.09] |
| High proportion of students held back | 0.28^***^ | [0.14,0.41] | 0.27^***^ | [0.14,0.41] | 0.27^***^ | [0.13,0.40] | 0.27^***^ | [0.14,0.41] | 0.27^***^ | [0.14,0.41] |
| High dropout rate | -0.28^***^ | [-0.39,-0.17] | -0.28^***^ | [-0.40,-0.17] | -0.28^***^ | [-0.39,-0.16] | -0.28^***^ | [-0.39,-0.17] | -0.28^***^ | [-0.40,-0.17] |
| Low attendance | -0.12 | [-0.28,0.03] | -0.12 | [-0.28,0.04] | -0.11 | [-0.27,0.04] | -0.13 | [-0.29,0.03] | -0.13 | [-0.28,0.03] |
| *Childhood adversities* |  |  |  |  |  |  |  |  |  |  |
| Run away from home | -0.29^**^ | [-0.47,-0.11] | -0.31^***^ | [-0.50,-0.13] | -0.29^**^ | [-0.47,-0.10] | -0.32^***^ | [-0.50,-0.13] | -0.31^***^ | [-0.50,-0.13] |
| Knife pulled at resp. | -0.53^***^ | [-0.75,-0.31] | -0.51^***^ | [-0.73,-0.29] | -0.50^***^ | [-0.72,-0.28] | -0.52^***^ | [-0.74,-0.30] | -0.52^***^ | [-0.74,-0.31] |
| Parents don’t care about resp. | -0.02 | [-0.29,0.25] | -0.09 | [-0.36,0.18] | -0.06 | [-0.33,0.21] | -0.07 | [-0.34,0.20] | -0.08 | [-0.35,0.20] |
| HH member touched in sexual way | 0.05 | [-0.14,0.23] | 0.04 | [-0.15,0.22] | 0.05 | [-0.14,0.24] | 0.03 | [-0.15,0.22] | 0.05 | [-0.14,0.23] |
| HH member kicked/hit/thrown | -0.09 | [-0.22,0.04] | -0.08 | [-0.22,0.05] | -0.09 | [-0.22,0.05] | -0.09 | [-0.22,0.04] | -0.08 | [-0.21,0.05] |
| HH received welfare during childhood | -0.61^***^ | [-0.73,-0.50] | -0.63^***^ | [-0.75,-0.51] | -0.61^***^ | [-0.73,-0.50] | -0.62^***^ | [-0.74,-0.51] | -0.62^***^ | [-0.74,-0.51] |
| No health insurance | -0.11 | [-0.27,0.06] | -0.09 | [-0.26,0.07] | -0.10 | [-0.27,0.06] | -0.10 | [-0.26,0.06] | -0.09 | [-0.25,0.07] |
| *Interview observations* |  |  |  |  |  |  |  |  |  |  |
| Evidence of drinking | -0.16 | [-0.43,0.11] | -0.16 | [-0.43,0.11] | -0.15 | [-0.42,0.12] | -0.16 | [-0.43,0.11] | -0.16 | [-0.43,0.11] |
| Safety concerns | -0.18 | [-0.43,0.07] | -0.19 | [-0.44,0.06] | -0.18 | [-0.43,0.08] | -0.19 | [-0.44,0.06] | -0.20 | [-0.46,0.05] |
| Poor dwelling condition | -0.05 | [-0.21,0.11] | -0.05 | [-0.21,0.11] | -0.05 | [-0.21,0.11] | -0.06 | [-0.22,0.10] | -0.06 | [-0.21,0.10] |
| Poor grooming | 0.09 | [-0.22,0.41] | 0.06 | [-0.25,0.38] | 0.10 | [-0.22,0.42] | 0.08 | [-0.24,0.40] | 0.04 | [-0.28,0.36] |
| *Health selection* |  |  |  |  |  |  |  |  |  |  |
| W1 equivalent measure | -0.03^**^ | [-0.05,-0.01] | -0.23^***^ | [-0.35,-0.10] | -0.34^***^ | [-0.48,-0.19] | -0.25 | [-0.53,0.03] | -0.11 | [-0.24,0.02] |
| Intercept | -0.96^***^ | [-1.43,-0.49] | -1.03^***^ | [-1.51,-0.56] | -0.96^***^ | [-1.43,-0.48] | -0.95^***^ | [-1.42,-0.48] | -0.92^***^ | [-1.39,-0.45] |
| AIC | 4001.65 |  | 3987.30 |  | 3983.99 |  | 4016.93 |  | 4007.10 |  |
| BIC | 4235.93 |  | 4221.38 |  | 4218.03 |  | 4251.23 |  | 4241.30 |  |
| pseudo R^2^ | 0.193 |  | 0.192 |  | 0.192 |  | 0.191 |  | 0.191 |  |
| Observations | 3495 |  | 3495 |  | 3495 |  | 3495 |  | 3495 |  |

Notes: 95% confidence intervals in brackets. ^*^ *p* < 0.05, ^**^ *p* < 0.01, ^***^ *p* < 0.001. *Source:* Add Health.

Table S3: Probit Regression Estimates Predicting College Completion, Males Only

|  | CESD |  | Binge |  | Painkiller |  | Hard drugs |  | Suicide |  |
| --- | --- | --- | --- | --- | --- | --- | --- | --- | --- | --- |
| *Demographics and IQ* |  |  |  |  |  |  |  |  |  |  |
| Age | -0.00 | [-0.04,0.03] | 0.02 | [-0.02,0.05] | 0.01 | [-0.03,0.04] | -0.00 | [-0.04,0.03] | -0.01 | [-0.04,0.03] |
| Hispanic | 0.32^***^ | [0.13,0.51] | 0.31^**^ | [0.12,0.50] | 0.31^**^ | [0.12,0.50] | 0.31^**^ | [0.13,0.50] | 0.32^***^ | [0.13,0.50] |
| Black(nonHisp) | 0.25^*^ | [0.05,0.45] | 0.20 | [-0.00,0.40] | 0.23^*^ | [0.02,0.43] | 0.24^*^ | [0.04,0.44] | 0.26^*^ | [0.06,0.46] |
| Asian(nonHisp) | 0.82^***^ | [0.56,1.08] | 0.78^***^ | [0.52,1.05] | 0.82^***^ | [0.56,1.09] | 0.81^***^ | [0.55,1.08] | 0.81^***^ | [0.55,1.08] |
| Other(nonHisp) | 0.93^**^ | [0.33,1.54] | 0.92^**^ | [0.31,1.53] | 0.90^**^ | [0.29,1.51] | 0.92^**^ | [0.32,1.53] | 0.94^**^ | [0.33,1.55] |
| Pbody test | 0.35^***^ | [0.28,0.42] | 0.35^***^ | [0.28,0.42] | 0.34^***^ | [0.27,0.41] | 0.35^***^ | [0.28,0.42] | 0.35^***^ | [0.28,0.41] |
| *Parents* |  |  |  |  |  |  |  |  |  |  |
| Occupation | 0.08^***^ | [0.03,0.12] | 0.07^***^ | [0.03,0.12] | 0.08^***^ | [0.03,0.12] | 0.08^***^ | [0.03,0.12] | 0.07^***^ | [0.03,0.12] |
| HH income | 0.23^***^ | [0.18,0.28] | 0.24^***^ | [0.19,0.29] | 0.24^***^ | [0.19,0.29] | 0.23^***^ | [0.18,0.28] | 0.23^***^ | [0.18,0.28] |
| Low edu. parents | -0.21 | [-0.44,0.01] | -0.21 | [-0.44,0.01] | -0.22 | [-0.44,0.01] | -0.22 | [-0.44,0.01] | -0.22 | [-0.44,0.00] |
| Married parents | -0.20^*^ | [-0.39,-0.01] | -0.24^*^ | [-0.43,-0.05] | -0.23^*^ | [-0.42,-0.04] | -0.21^*^ | [-0.40,-0.02] | -0.20^*^ | [-0.39,-0.01] |
| Single parent at age=0 | -0.20^*^ | [-0.36,-0.04] | -0.20^*^ | [-0.36,-0.04] | -0.20^*^ | [-0.36,-0.04] | -0.21^*^ | [-0.37,-0.05] | -0.21^*^ | [-0.37,-0.05] |
| Single parent at age=13 | -0.13 | [-0.29,0.04] | -0.12 | [-0.29,0.05] | -0.12 | [-0.29,0.05] | -0.12 | [-0.29,0.04] | -0.13 | [-0.29,0.04] |
| Parent in jail | -0.33^***^ | [-0.53,-0.14] | -0.33^***^ | [-0.52,-0.14] | -0.34^***^ | [-0.53,-0.14] | -0.34^***^ | [-0.53,-0.14] | -0.33^***^ | [-0.52,-0.14] |
| Parent binges | -0.06 | [-0.23,0.12] | -0.02 | [-0.20,0.15] | -0.04 | [-0.22,0.14] | -0.05 | [-0.23,0.12] | -0.06 | [-0.24,0.12] |
| Parents talk about separation | -0.25^*^ | [-0.45,-0.05] | -0.25^*^ | [-0.45,-0.05] | -0.24^*^ | [-0.44,-0.04] | -0.25^*^ | [-0.45,-0.05] | -0.25^*^ | [-0.45,-0.05] |
| Parents fight at all | -0.11 | [-0.25,0.04] | -0.10 | [-0.24,0.05] | -0.10 | [-0.25,0.05] | -0.10 | [-0.25,0.04] | -0.11 | [-0.26,0.04] |
| Parents fight a lot | 0.50^*^ | [0.09,0.91] | 0.51^*^ | [0.10,0.92] | 0.49^*^ | [0.08,0.90] | 0.49^*^ | [0.08,0.90] | 0.49^*^ | [0.08,0.90] |
| *Neighborhood* |  |  |  |  |  |  |  |  |  |  |
| Low proportion White | -0.12 | [-0.29,0.06] | -0.13 | [-0.31,0.04] | -0.11 | [-0.29,0.07] | -0.12 | [-0.30,0.05] | -0.11 | [-0.29,0.06] |
|  |  |  |  |  |  |  |  |  |  |  |
| Low median HH income | 0.11 | [-0.06,0.28] | 0.12 | [-0.05,0.28] | 0.11 | [-0.05,0.28] | 0.12 | [-0.05,0.28] | 0.11 | [-0.05,0.28] |
| High unemployment | 0.06 | [-0.11,0.23] | 0.04 | [-0.13,0.21] | 0.06 | [-0.11,0.23] | 0.05 | [-0.12,0.22] | 0.05 | [-0.12,0.22] |
| *School* |  |  |  |  |  |  |  |  |  |  |
| Large class size | 0.01 | [-0.14,0.15] | 0.01 | [-0.14,0.16] | 0.00 | [-0.14,0.15] | 0.01 | [-0.14,0.16] | 0.01 | [-0.13,0.16] |
| Low ratio of teachers with masters | -0.04 | [-0.21,0.12] | -0.05 | [-0.22,0.11] | -0.06 | [-0.22,0.10] | -0.04 | [-0.20,0.12] | -0.04 | [-0.20,0.12] |
| High proportion of students held back | 0.07 | [-0.10,0.24] | 0.06 | [-0.11,0.23] | 0.06 | [-0.11,0.23] | 0.07 | [-0.09,0.24] | 0.07 | [-0.10,0.24] |
| High dropout rate | -0.18^**^ | [-0.31,-0.04] | -0.17^*^ | [-0.31,-0.04] | -0.17^*^ | [-0.31,-0.04] | -0.18^**^ | [-0.31,-0.05] | -0.18^**^ | [-0.31,-0.04] |
| Low attendance | -0.15 | [-0.33,0.04] | -0.13 | [-0.32,0.05] | -0.14 | [-0.32,0.05] | -0.15 | [-0.33,0.04] | -0.15 | [-0.33,0.04] |
| *Childhood adversities* |  |  |  |  |  |  |  |  |  |  |
| Run away from home | -0.19 | [-0.43,0.05] | -0.18 | [-0.42,0.06] | -0.15 | [-0.39,0.09] | -0.18 | [-0.42,0.06] | -0.22 | [-0.46,0.02] |
| Knife pulled at resp. | -0.39^***^ | [-0.54,-0.24] | -0.36^***^ | [-0.51,-0.21] | -0.33^***^ | [-0.49,-0.18] | -0.39^***^ | [-0.54,-0.24] | -0.41^***^ | [-0.56,-0.26] |
| Parents don’t care about resp. | -0.11 | [-0.46,0.24] | -0.13 | [-0.48,0.21] | -0.12 | [-0.47,0.23] | -0.13 | [-0.47,0.22] | -0.16 | [-0.51,0.19] |
| HH member touched in sexual way | -0.27 | [-0.68,0.14] | -0.26 | [-0.68,0.15] | -0.27 | [-0.69,0.15] | -0.27 | [-0.68,0.15] | -0.25 | [-0.67,0.16] |
| HH member kicked/hit/thrown | -0.05 | [-0.20,0.09] | -0.06 | [-0.20,0.09] | -0.05 | [-0.20,0.09] | -0.05 | [-0.20,0.09] | -0.06 | [-0.21,0.08] |
| HH received welfare during childhood | -0.51^***^ | [-0.68,-0.34] | -0.51^***^ | [-0.68,-0.34] | -0.50^***^ | [-0.67,-0.33] | -0.51^***^ | [-0.68,-0.34] | -0.51^***^ | [-0.68,-0.34] |
| No health insurance | -0.22 | [-0.44,0.01] | -0.22 | [-0.45,0.00] | -0.24^*^ | [-0.47,-0.01] | -0.22 | [-0.44,0.01] | -0.22 | [-0.45,0.00] |
| *Interview observations* |  |  |  |  |  |  |  |  |  |  |
| Evidence of drinking | -0.07 | [-0.38,0.23] | -0.05 | [-0.36,0.26] | -0.06 | [-0.37,0.25] | -0.07 | [-0.38,0.24] | -0.06 | [-0.37,0.24] |
| Safety concerns | 0.20 | [-0.11,0.51] | 0.17 | [-0.14,0.48] | 0.19 | [-0.12,0.50] | 0.19 | [-0.12,0.50] | 0.18 | [-0.13,0.49] |
| Poor dwelling condition | -0.39^***^ | [-0.61,-0.18] | -0.40^***^ | [-0.62,-0.19] | -0.40^***^ | [-0.62,-0.19] | -0.39^***^ | [-0.61,-0.18] | -0.39^***^ | [-0.61,-0.18] |
| Poor grooming | -0.27 | [-0.63,0.08] | -0.22 | [-0.57,0.14] | -0.21 | [-0.57,0.14] | -0.26 | [-0.61,0.10] | -0.28 | [-0.63,0.07] |
| *Health selection* |  |  |  |  |  |  |  |  |  |  |
| W1 equivalent measure | -0.01 | [-0.04,0.01] | -0.26^***^ | [-0.40,-0.12] | -0.31^***^ | [-0.47,-0.14] | -0.17 | [-0.43,0.09] | 0.12 | [-0.06,0.30] |
| Intercept | -0.86^**^ | [-1.41,-0.30] | -1.11^***^ | [-1.68,-0.53] | -0.98^***^ | [-1.54,-0.42] | -0.88^**^ | [-1.43,-0.32] | -0.82^**^ | [-1.38,-0.27] |
| AIC | 2967.62 |  | 2941.30 |  | 2935.69 |  | 2968.12 |  | 2965.58 |  |
| BIC | 3191.62 |  | 3165.09 |  | 3159.22 |  | 3192.13 |  | 3189.50 |  |
| Pseudo R^2^ | 0.188 |  | 0.191 |  | 0.189 |  | 0.188 |  | 0.188 |  |
| Observations | 2650 |  | 2650 |  | 2650 |  | 2650 |  | 2650 |  |

Notes: 95% confidence intervals in brackets. ^*^ *p* < 0.05, ^**^ *p* < 0.01, ^***^ *p* < 0.001. *Source:* Add Health.

Table S4: Probit Regression Estimates Predicting College Completion, Whites Only

|  | CESD |  | Binge |  | Painkiller |  | Hard drugs |  | Suicide |  |
| --- | --- | --- | --- | --- | --- | --- | --- | --- | --- | --- |
| *Demographics and IQ* |  |  |  |  |  |  |  |  |  |  |
| Age | 0.01 | [-0.02,0.04] | 0.02 | [-0.01,0.05] | 0.01 | [-0.02,0.04] | 0.01 | [-0.02,0.04] | 0.00 | [-0.02,0.03] |
| Male | -0.40^***^ | [-0.49,-0.30] | -0.37^***^ | [-0.46,-0.27] | -0.38^***^ | [-0.47,-0.29] | -0.38^***^ | [-0.47,-0.28] | -0.38^***^ | [-0.47,-0.29] |
| Pbody test | 0.36^***^ | [0.30,0.42] | 0.36^***^ | [0.30,0.42] | 0.36^***^ | [0.30,0.42] | 0.37^***^ | [0.31,0.43] | 0.36^***^ | [0.30,0.43] |
| *Parents* |  |  |  |  |  |  |  |  |  |  |
| Occupation | 0.10^***^ | [0.07,0.14] | 0.10^***^ | [0.06,0.13] | 0.10^***^ | [0.06,0.13] | 0.10^***^ | [0.07,0.14] | 0.10^***^ | [0.07,0.14] |
| HH income | 0.21^***^ | [0.17,0.25] | 0.22^***^ | [0.17,0.26] | 0.22^***^ | [0.17,0.26] | 0.21^***^ | [0.17,0.25] | 0.21^***^ | [0.17,0.25] |
| Low edu. parents | -0.56^***^ | [-0.80,-0.33] | -0.56^***^ | [-0.79,-0.33] | -0.57^***^ | [-0.80,-0.34] | -0.57^***^ | [-0.80,-0.34] | -0.57^***^ | [-0.80,-0.33] |
| Married parents | -0.02 | [-0.19,0.14] | -0.05 | [-0.21,0.12] | -0.04 | [-0.20,0.13] | -0.02 | [-0.19,0.14] | -0.02 | [-0.18,0.15] |
| Single parent at age=0 | -0.24^***^ | [-0.39,-0.10] | -0.25^***^ | [-0.39,-0.10] | -0.25^***^ | [-0.39,-0.11] | -0.25^***^ | [-0.39,-0.11] | -0.25^***^ | [-0.39,-0.11] |
| Single parent at age=13 | 0.04 | [-0.11,0.18] | 0.03 | [-0.12,0.18] | 0.05 | [-0.10,0.20] | 0.04 | [-0.11,0.18] | 0.04 | [-0.11,0.18] |
| Parent in jail | -0.32^***^ | [-0.48,-0.16] | -0.32^***^ | [-0.48,-0.16] | -0.30^***^ | [-0.46,-0.14] | -0.32^***^ | [-0.48,-0.16] | -0.32^***^ | [-0.48,-0.16] |
| Parent binges | -0.02 | [-0.16,0.12] | 0.01 | [-0.13,0.15] | -0.02 | [-0.16,0.12] | -0.02 | [-0.16,0.12] | -0.02 | [-0.16,0.12] |
| Parents talk about separation | -0.22^**^ | [-0.38,-0.05] | -0.21^*^ | [-0.37,-0.05] | -0.20^*^ | [-0.37,-0.04] | -0.22^**^ | [-0.38,-0.06] | -0.22^**^ | [-0.38,-0.05] |
| Parents fight at all | -0.06 | [-0.18,0.06] | -0.06 | [-0.18,0.06] | -0.05 | [-0.18,0.07] | -0.06 | [-0.18,0.06] | -0.06 | [-0.18,0.06] |
| Parents fight a lot | 0.10 | [-0.25,0.45] | 0.09 | [-0.26,0.44] | 0.07 | [-0.29,0.42] | 0.08 | [-0.27,0.43] | 0.08 | [-0.27,0.43] |
|  |  |  |  |  |  |  |  |  |  |  |
| *Neighborhood* |  |  |  |  |  |  |  |  |  |  |
| HH received welfare during childhood | -0.59^***^ | [-0.72,-0.46] | -0.61^***^ | [-0.74,-0.48] | -0.59^***^ | [-0.72,-0.47] | -0.60^***^ | [-0.72,-0.47] | -0.60^***^ | [-0.72,-0.47] |
| Low proportion White | -0.00 | [-0.18,0.18] | -0.01 | [-0.19,0.17] | -0.00 | [-0.18,0.18] | -0.01 | [-0.18,0.17] | -0.00 | [-0.18,0.18] |
| Low median HH income | -0.02 | [-0.17,0.13] | -0.02 | [-0.17,0.13] | -0.02 | [-0.17,0.13] | -0.01 | [-0.16,0.13] | -0.01 | [-0.16,0.14] |
| High unemployment | -0.05 | [-0.20,0.10] | -0.05 | [-0.20,0.10] | -0.06 | [-0.21,0.10] | -0.05 | [-0.20,0.10] | -0.06 | [-0.21,0.10] |
| *School* |  |  |  |  |  |  |  |  |  |  |
| Large class size | 0.04 | [-0.09,0.16] | 0.03 | [-0.10,0.16] | 0.03 | [-0.09,0.16] | 0.05 | [-0.08,0.17] | 0.04 | [-0.09,0.16] |
| Low ratio of teachers with masters | -0.07 | [-0.21,0.06] | -0.07 | [-0.21,0.06] | -0.08 | [-0.21,0.06] | -0.07 | [-0.20,0.07] | -0.07 | [-0.21,0.06] |
| High proportion of students held back | 0.22^**^ | [0.07,0.38] | 0.21^**^ | [0.05,0.37] | 0.21^**^ | [0.05,0.37] | 0.22^**^ | [0.06,0.38] | 0.22^**^ | [0.06,0.37] |
| High dropout rate | -0.26^***^ | [-0.37,-0.16] | -0.27^***^ | [-0.37,-0.16] | -0.26^***^ | [-0.37,-0.15] | -0.27^***^ | [-0.37,-0.16] | -0.27^***^ | [-0.37,-0.16] |
| Low attendance | -0.11 | [-0.30,0.09] | -0.10 | [-0.30,0.09] | -0.09 | [-0.28,0.10] | -0.11 | [-0.30,0.08] | -0.11 | [-0.30,0.08] |
| *Childhood adversities* |  |  |  |  |  |  |  |  |  |  |
| Run away from home | -0.26^**^ | [-0.45,-0.07] | -0.28^**^ | [-0.47,-0.09] | -0.26^**^ | [-0.45,-0.07] | -0.27^**^ | [-0.46,-0.08] | -0.29^**^ | [-0.48,-0.10] |
| Knife pulled at resp. | -0.37^***^ | [-0.53,-0.20] | -0.36^***^ | [-0.52,-0.19] | -0.34^***^ | [-0.50,-0.17] | -0.37^***^ | [-0.54,-0.21] | -0.38^***^ | [-0.55,-0.21] |
| Parents don’t care about resp. | 0.02 | [-0.27,0.32] | -0.04 | [-0.34,0.26] | -0.03 | [-0.33,0.27] | -0.03 | [-0.32,0.27] | -0.05 | [-0.35,0.25] |
| HH member touched in sexual way | -0.05 | [-0.27,0.17] | -0.06 | [-0.29,0.16] | -0.04 | [-0.26,0.18] | -0.05 | [-0.27,0.17] | -0.05 | [-0.27,0.17] |
| HH member kicked/hit/thrown | -0.10 | [-0.22,0.03] | -0.11 | [-0.23,0.02] | -0.11 | [-0.23,0.02] | -0.10 | [-0.23,0.02] | -0.10 | [-0.23,0.02] |
| No health insurance | -0.30^**^ | [-0.50,-0.10] | -0.29^**^ | [-0.49,-0.09] | -0.32^**^ | [-0.52,-0.12] | -0.29^**^ | [-0.50,-0.09] | -0.29^**^ | [-0.49,-0.09] |
| *Interview observations* |  |  |  |  |  |  |  |  |  |  |
| Evidence of drinking | -0.10 | [-0.38,0.17] | -0.10 | [-0.37,0.18] | -0.10 | [-0.38,0.18] | -0.10 | [-0.38,0.17] | -0.10 | [-0.37,0.18] |
| Safety concerns | 0.02 | [-0.30,0.33] | -0.02 | [-0.34,0.29] | -0.01 | [-0.32,0.31] | -0.01 | [-0.33,0.31] | -0.02 | [-0.34,0.30] |
| Poor dwelling condition | -0.21^*^ | [-0.38,-0.04] | -0.21^*^ | [-0.38,-0.04] | -0.21^*^ | [-0.38,-0.04] | -0.21^*^ | [-0.38,-0.04] | -0.21^*^ | [-0.38,-0.04] |
| Poor grooming | -0.18 | [-0.50,0.15] | -0.18 | [-0.50,0.15] | -0.16 | [-0.49,0.17] | -0.16 | [-0.49,0.17] | -0.19 | [-0.52,0.13] |
| *Health selection* |  |  |  |  |  |  |  |  |  |  |
| W1 equivalent measure | -0.03^**^ | [-0.06,-0.01] | -0.23^***^ | [-0.34,-0.12] | -0.27^***^ | [-0.40,-0.14] | -0.26^*^ | [-0.48,-0.03] | -0.05 | [-0.18,0.08] |
| Intercept | -0.79^***^ | [-1.25,-0.33] | -0.99^***^ | [-1.46,-0.51] | -0.84^***^ | [-1.30,-0.37] | -0.82^***^ | [-1.29,-0.36] | -0.78^***^ | [-1.24,-0.32] |
| AIC | 4290.98 |  | 4262.31 |  | 4262.37 |  | 4296.67 |  | 4293.83 |  |
| BIC | 4510.18 |  | 4481.30 |  | 4481.26 |  | 4515.87 |  | 4512.97 |  |
| pseudo R^2^ | 0.209 |  | 0.210 |  | 0.208 |  | 0.208 |  | 0.207 |  |
| Observations | 3843 |  | 3843 |  | 3843 |  | 3843 |  | 3843 |  |

Notes: 95% confidence intervals in brackets. ^*^ *p* < 0.05, ^**^ *p* < 0.01, ^***^ *p* < 0.001. *Source:* Add Health.

Table S5: Probit Regression Estimates Predicting College Completion, Hispanics Only

|  | CESD |  | Binge |  | Painkiller |  | Hard drugs |  | Suicide |  |
| --- | --- | --- | --- | --- | --- | --- | --- | --- | --- | --- |
| *Demographics and IQ* |  |  |  |  |  |  |  |  |  |  |
| Age | 0.03 | [-0.04,0.09] | 0.03 | [-0.03,0.10] | 0.03 | [-0.03,0.10] | 0.03 | [-0.04,0.09] | 0.02 | [-0.04,0.09] |
| Male | -0.28^**^ | [-0.48,-0.08] | -0.26^**^ | [-0.45,-0.06] | -0.26^**^ | [-0.46,-0.06] | -0.27^**^ | [-0.46,-0.08] | -0.26^**^ | [-0.46,-0.07] |
| Pbody test | 0.28^***^ | [0.18,0.39] | 0.25^***^ | [0.15,0.35] | 0.25^***^ | [0.15,0.35] | 0.26^***^ | [0.16,0.36] | 0.25^***^ | [0.15,0.35] |
| *Parents* |  |  |  |  |  |  |  |  |  |  |
| Occupation | 0.08 | [-0.00,0.16] | 0.08^*^ | [0.00,0.16] | 0.09^*^ | [0.01,0.17] | 0.08^*^ | [0.00,0.16] | 0.08^*^ | [0.00,0.16] |
| HH income | 0.10^*^ | [0.01,0.19] | 0.10^*^ | [0.01,0.19] | 0.09^*^ | [0.00,0.18] | 0.09^*^ | [0.01,0.18] | 0.09^*^ | [0.00,0.18] |
| Low edu. parents | -0.02 | [-0.25,0.21] | -0.06 | [-0.29,0.17] | -0.06 | [-0.29,0.17] | -0.05 | [-0.28,0.18] | -0.05 | [-0.27,0.18] |
| Married parents | -0.30^*^ | [-0.59,-0.01] | -0.29^*^ | [-0.58,-0.00] | -0.30^*^ | [-0.59,-0.01] | -0.30^*^ | [-0.59,-0.01] | -0.29^*^ | [-0.58,-0.00] |
| Single parent at age=0 | -0.05 | [-0.30,0.20] | -0.05 | [-0.30,0.20] | -0.07 | [-0.33,0.18] | -0.07 | [-0.32,0.19] | -0.06 | [-0.32,0.19] |
| Single parent at age=13 | -0.02 | [-0.30,0.26] | -0.01 | [-0.29,0.27] | 0.01 | [-0.27,0.29] | -0.01 | [-0.29,0.27] | -0.02 | [-0.30,0.26] |
| Parent in jail | -0.01 | [-0.28,0.27] | 0.01 | [-0.27,0.28] | 0.00 | [-0.28,0.28] | -0.01 | [-0.28,0.27] | -0.02 | [-0.30,0.26] |
| Parent binges | -0.08 | [-0.37,0.21] | -0.09 | [-0.38,0.20] | -0.10 | [-0.40,0.19] | -0.10 | [-0.39,0.19] | -0.09 | [-0.38,0.20] |
| Parents talk about separation | -0.09 | [-0.40,0.22] | -0.08 | [-0.39,0.23] | -0.05 | [-0.37,0.26] | -0.08 | [-0.39,0.23] | -0.10 | [-0.41,0.21] |
| Parents fight at all | 0.13 | [-0.13,0.39] | 0.14 | [-0.12,0.40] | 0.17 | [-0.09,0.43] | 0.15 | [-0.11,0.41] | 0.15 | [-0.11,0.41] |
| Parents fight a lot | 0.17 | [-0.40,0.73] | 0.16 | [-0.40,0.72] | 0.15 | [-0.43,0.72] | 0.11 | [-0.45,0.68] | 0.14 | [-0.42,0.70] |
| *Neighborhood* |  |  |  |  |  |  |  |  |  |  |
| Low proportion White | -0.15 | [-0.40,0.10] | -0.15 | [-0.40,0.09] | -0.16 | [-0.41,0.09] | -0.17 | [-0.41,0.08] | -0.16 | [-0.40,0.09] |
| Low median HH income | 0.22 | [-0.05,0.49] | 0.18 | [-0.09,0.45] | 0.19 | [-0.08,0.46] | 0.20 | [-0.06,0.47] | 0.19 | [-0.08,0.46] |
| High unemployment | -0.11 | [-0.35,0.13] | -0.11 | [-0.35,0.13] | -0.11 | [-0.35,0.13] | -0.11 | [-0.35,0.13] | -0.11 | [-0.35,0.13] |
| *School* |  |  |  |  |  |  |  |  |  |  |
| Large class size | 0.08 | [-0.22,0.39] | 0.06 | [-0.25,0.37] | 0.05 | [-0.25,0.36] | 0.07 | [-0.23,0.38] | 0.08 | [-0.23,0.38] |
| Low ratio of teachers with masters | -0.06 | [-0.36,0.24] | -0.05 | [-0.35,0.24] | -0.08 | [-0.38,0.22] | -0.05 | [-0.34,0.25] | -0.05 | [-0.35,0.24] |
| High proportion of students held back | 0.21 | [-0.04,0.46] | 0.20 | [-0.06,0.46] | 0.19 | [-0.07,0.44] | 0.20 | [-0.05,0.46] | 0.20 | [-0.05,0.46] |
| High dropout rate | -0.11 | [-0.38,0.17] | -0.07 | [-0.35,0.20] | -0.09 | [-0.37,0.18] | -0.08 | [-0.35,0.20] | -0.08 | [-0.36,0.19] |
| Low attendance | 0.02 | [-0.30,0.35] | 0.04 | [-0.29,0.37] | 0.06 | [-0.27,0.39] | 0.03 | [-0.30,0.36] | 0.03 | [-0.30,0.36] |
| *Childhood adversities* |  |  |  |  |  |  |  |  |  |  |
| Run away from home | -0.32 | [-0.67,0.03] | -0.30 | [-0.66,0.06] | -0.27 | [-0.63,0.09] | -0.33 | [-0.69,0.02] | -0.34 | [-0.70,0.02] |
| Knife pulled at resp. | -0.52^**^ | [-0.83,-0.20] | -0.48^**^ | [-0.80,-0.17] | -0.48^**^ | [-0.79,-0.16] | -0.51^**^ | [-0.83,-0.20] | -0.52^**^ | [-0.83,-0.21] |
| Parents don’t care about resp. | -0.20 | [-0.74,0.35] | -0.27 | [-0.82,0.29] | -0.12 | [-0.68,0.45] | -0.20 | [-0.74,0.35] | -0.27 | [-0.83,0.29] |
| HH member touched in sexual way | 0.51^*^ | [0.07,0.94] | 0.49^*^ | [0.06,0.92] | 0.48^*^ | [0.04,0.91] | 0.48^*^ | [0.05,0.91] | 0.49^*^ | [0.06,0.93] |
| HH member kicked/hit/thrown | -0.02 | [-0.29,0.24] | 0.01 | [-0.25,0.27] | 0.02 | [-0.24,0.28] | 0.01 | [-0.25,0.27] | 0.01 | [-0.25,0.27] |
| HH received welfare during childhood | -0.42^**^ | [-0.68,-0.15] | -0.42^**^ | [-0.69,-0.16] | -0.42^**^ | [-0.68,-0.16] | -0.44^**^ | [-0.70,-0.17] | -0.46^***^ | [-0.72,-0.20] |
| No health insurance | -0.08 | [-0.34,0.17] | -0.07 | [-0.32,0.19] | -0.08 | [-0.33,0.18] | -0.07 | [-0.32,0.19] | -0.06 | [-0.31,0.20] |
| *Interview observations* |  |  |  |  |  |  |  |  |  |  |
| Evidence of drinking | -0.00 | [-0.51,0.51] | -0.02 | [-0.53,0.49] | 0.05 | [-0.46,0.56] | -0.01 | [-0.52,0.49] | -0.03 | [-0.53,0.48] |
| Safety concerns | -0.27 | [-0.69,0.14] | -0.29 | [-0.70,0.13] | -0.26 | [-0.67,0.16] | -0.26 | [-0.68,0.15] | -0.27 | [-0.68,0.15] |
| Poor dwelling condition | -0.52^**^ | [-0.87,-0.17] | -0.51^**^ | [-0.87,-0.16] | -0.53^**^ | [-0.89,-0.18] | -0.52^**^ | [-0.87,-0.17] | -0.51^**^ | [-0.86,-0.16] |
| Poor grooming | 0.03 | [-0.59,0.65] | 0.10 | [-0.53,0.74] | 0.05 | [-0.57,0.67] | 0.03 | [-0.59,0.65] | -0.08 | [-0.73,0.57] |
| *Health selection* |  |  |  |  |  |  |  |  |  |  |
| W1 equivalent measure | -0.02 | [-0.06,0.03] | -0.19 | [-0.44,0.07] | -0.43^**^ | [-0.74,-0.12] | -0.26 | [-0.74,0.23] | 0.01 | [-0.27,0.30] |
| Intercept | -0.73 | [-1.78,0.31] | -0.80 | [-1.87,0.26] | -0.81 | [-1.85,0.24] | -0.72 | [-1.76,0.32] | -0.71 | [-1.75,0.33] |
| AIC | 1030.45 |  | 1029.76 |  | 1025.45 |  | 1036.14 |  | 1034.2 |  |
| BIC | 1196.82 |  | 1195.89 |  | 1191.58 |  | 1202.59 |  | 1200.6 |  |
| Pseudo R^2^ | 0.136 |  | 0.131 |  | 0.135 |  | 0.133 |  | 0.130 |  |
| Observations | 851 |  | 851 |  | 851 |  | 851 |  | 851 |  |

Notes: 95% confidence intervals in brackets. ^*^ *p* < 0.05, ^**^ *p* < 0.01, ^***^ *p* < 0.001. *Source:* Add Health.

Table S6: Probit Regression Estimates Predicting College Completion, Blacks Only

| *Demographics and IQ* | CESD |  | Binge |  | Painkiller |  | Hard drugs |  | Suicide |  |
| --- | --- | --- | --- | --- | --- | --- | --- | --- | --- | --- |
|  |  |  |  |  |  |  |  |  |  |  |
| Age | 0.04 | [-0.01,0.10] | 0.05 | [-0.01,0.10] | 0.04 | [-0.01,0.10] | 0.04 | [-0.01,0.10] | 0.04 | [-0.01,0.10] |
| Male | -0.66^***^ | [-0.85,-0.47] | -0.66^***^ | [-0.86,-0.47] | -0.64^***^ | [-0.84,-0.45] | -0.67^***^ | [-0.86,-0.47] | -0.65^***^ | [-0.85,-0.46] |
| Pbody test | 0.37^***^ | [0.27,0.47] | 0.38^***^ | [0.28,0.48] | 0.37^***^ | [0.26,0.47] | 0.38^***^ | [0.28,0.49] | 0.37^***^ | [0.27,0.48] |
| Occupation | 0.11^**^ | [0.04,0.17] | 0.11^**^ | [0.04,0.18] | 0.11^**^ | [0.04,0.18] | 0.11^**^ | [0.04,0.18] | 0.11^**^ | [0.04,0.17] |
| HH income | 0.08^*^ | [0.01,0.16] | 0.08^*^ | [0.00,0.16] | 0.08^*^ | [0.01,0.16] | 0.08^*^ | [0.00,0.16] | 0.08^*^ | [0.00,0.16] |
| Low edu. parents | -0.30 | [-0.61,0.01] | -0.29 | [-0.60,0.01] | -0.30 | [-0.61,0.01] | -0.30 | [-0.60,0.01] | -0.30 | [-0.61,0.01] |
| Married parents | -0.20 | [-0.45,0.06] | -0.20 | [-0.46,0.06] | -0.21 | [-0.47,0.05] | -0.21 | [-0.47,0.05] | -0.19 | [-0.45,0.07] |
| Single parent at age=0 | -0.16 | [-0.35,0.04] | -0.16 | [-0.35,0.04] | -0.16 | [-0.35,0.04] | -0.17 | [-0.37,0.02] | -0.15 | [-0.35,0.04] |
| Single parent at age=13 | -0.15 | [-0.37,0.07] | -0.15 | [-0.36,0.07] | -0.15 | [-0.37,0.07] | -0.16 | [-0.38,0.06] | -0.15 | [-0.37,0.07] |
| Parent in jail | -0.13 | [-0.37,0.11] | -0.13 | [-0.36,0.11] | -0.13 | [-0.37,0.10] | -0.13 | [-0.36,0.11] | -0.13 | [-0.37,0.10] |
| Parent binges | -0.17 | [-0.46,0.11] | -0.17 | [-0.45,0.11] | -0.17 | [-0.45,0.12] | -0.18 | [-0.47,0.10] | -0.18 | [-0.46,0.10] |
| Parents talk about separation | 0.18 | [-0.12,0.47] | 0.17 | [-0.12,0.47] | 0.19 | [-0.11,0.48] | 0.16 | [-0.13,0.46] | 0.19 | [-0.11,0.49] |
| Parents fight at all | -0.02 | [-0.26,0.21] | -0.02 | [-0.26,0.21] | -0.03 | [-0.27,0.20] | -0.02 | [-0.26,0.21] | -0.04 | [-0.27,0.20] |
| Parents fight a lot | -0.10 | [-0.68,0.48] | -0.11 | [-0.69,0.47] | -0.11 | [-0.69,0.46] | -0.12 | [-0.70,0.46] | -0.09 | [-0.67,0.50] |
| *Neighborhood* |  |  |  |  |  |  |  |  |  |  |
| Low proportion White | -0.11 | [-0.35,0.12] | -0.13 | [-0.36,0.11] | -0.11 | [-0.35,0.12] | -0.11 | [-0.35,0.12] | -0.11 | [-0.34,0.13] |
| Low median HH income | -0.04 | [-0.25,0.16] | -0.02 | [-0.23,0.19] | -0.05 | [-0.26,0.16] | -0.04 | [-0.25,0.16] | -0.04 | [-0.25,0.16] |
| High unemployment | 0.14 | [-0.07,0.34] | 0.13 | [-0.08,0.33] | 0.14 | [-0.06,0.35] | 0.14 | [-0.06,0.34] | 0.14 | [-0.07,0.34] |
| *School* |  |  |  |  |  |  |  |  |  |  |
| Large class size | -0.13 | [-0.34,0.09] | -0.12 | [-0.33,0.10] | -0.13 | [-0.34,0.09] | -0.12 | [-0.34,0.09] | -0.12 | [-0.34,0.09] |
| Low ratio of teachers with masters | 0.04 | [-0.20,0.27] | 0.04 | [-0.20,0.28] | 0.05 | [-0.19,0.29] | 0.05 | [-0.19,0.28] | 0.03 | [-0.21,0.27] |
| High proportion of students held back | -0.07 | [-0.29,0.16] | -0.08 | [-0.30,0.15] | -0.07 | [-0.30,0.16] | -0.07 | [-0.30,0.16] | -0.07 | [-0.29,0.16] |
| High dropout rate | -0.20 | [-0.42,0.02] | -0.21 | [-0.43,0.01] | -0.19 | [-0.41,0.03] | -0.20 | [-0.42,0.02] | -0.20 | [-0.42,0.02] |
| Low attendance | -0.07 | [-0.29,0.16] | -0.05 | [-0.28,0.17] | -0.07 | [-0.30,0.15] | -0.06 | [-0.28,0.17] | -0.07 | [-0.29,0.16] |
| *Childhood adversities* |  |  |  |  |  |  |  |  |  |  |
| Run away from home | -0.36^*^ | [-0.72,-0.01] | -0.41^*^ | [-0.77,-0.05] | -0.36^*^ | [-0.72,-0.01] | -0.40^*^ | [-0.75,-0.04] | -0.37^*^ | [-0.72,-0.01] |
| Knife pulled at resp. | -0.47^***^ | [-0.73,-0.21] | -0.44^***^ | [-0.71,-0.18] | -0.44^**^ | [-0.71,-0.18] | -0.50^***^ | [-0.76,-0.23] | -0.48^***^ | [-0.74,-0.21] |
| Parents don’t care about resp. | -0.06 | [-0.52,0.40] | -0.10 | [-0.56,0.36] | -0.10 | [-0.56,0.35] | -0.11 | [-0.58,0.35] | -0.07 | [-0.53,0.40] |
| HH member touched in sexual way | -0.19 | [-0.60,0.23] | -0.18 | [-0.59,0.24] | -0.17 | [-0.59,0.24] | -0.19 | [-0.61,0.22] | -0.17 | [-0.59,0.25] |
| HH member kicked/hit/thrown | 0.03 | [-0.20,0.26] | 0.03 | [-0.20,0.26] | 0.03 | [-0.20,0.26] | 0.02 | [-0.21,0.25] | 0.02 | [-0.21,0.25] |
| HH received welfare during childhood | -0.63^***^ | [-0.82,-0.43] | -0.61^***^ | [-0.81,-0.42] | -0.62^***^ | [-0.81,-0.42] | -0.63^***^ | [-0.82,-0.43] | -0.63^***^ | [-0.82,-0.43] |
| No health insurance | -0.13 | [-0.42,0.17] | -0.13 | [-0.43,0.17] | -0.13 | [-0.42,0.17] | -0.14 | [-0.43,0.16] | -0.13 | [-0.43,0.16] |
| *Interview observations* |  |  |  |  |  |  |  |  |  |  |
| Evidence of drinking | -0.11 | [-0.54,0.32] | -0.11 | [-0.54,0.33] | -0.11 | [-0.54,0.32] | -0.11 | [-0.54,0.32] | -0.12 | [-0.55,0.31] |
| Safety concerns | -0.27 | [-0.62,0.08] | -0.27 | [-0.62,0.07] | -0.25 | [-0.60,0.10] | -0.27 | [-0.62,0.08] | -0.28 | [-0.63,0.07] |
| Poor dwelling condition | 0.11 | [-0.16,0.37] | 0.09 | [-0.17,0.36] | 0.09 | [-0.18,0.36] | 0.10 | [-0.17,0.36] | 0.10 | [-0.17,0.36] |
| Poor grooming | 0.11 | [-0.36,0.58] | 0.13 | [-0.34,0.61] | 0.19 | [-0.29,0.68] | 0.12 | [-0.35,0.58] | 0.10 | [-0.37,0.57] |
| *Health selection* |  |  |  |  |  |  |  |  |  |  |
| W1 equivalent measure | -0.02 | [-0.06,0.02] | -0.19 | [-0.48,0.10] | -0.16 | [-0.45,0.13] | 0.79 | [-0.06,1.63] | -0.11 | [-0.38,0.15] |
| Intercept | -0.38 | [-1.25,0.49] | -0.47 | [-1.35,0.41] | -0.38 | [-1.26,0.50] | -0.35 | [-1.22,0.52] | -0.37 | [-1.24,0.50] |
| AIC | 1227.71 |  | 1221.93 |  | 1219.95 |  | 1225.29 |  | 1225.84 |  |
| BIC | 1402.62 |  | 1396.75 |  | 1394.42 |  | 1400.24 |  | 1400.56 |  |
| Pseudo R^2^ | 0.212 |  | 0.214 |  | 0.209 |  | 0.214 |  | 0.210 |  |
| Observations | 1080 |  | 1080 |  | 1080 |  | 1080 |  | 1080 |  |

Notes: 95% confidence intervals in brackets. ^*^ *p* < 0.05, ^**^ *p* < 0.01, ^***^ *p* < 0.001. *Source:* Add Health.

Table S7: Homogeneous Effect of College Completion on Selected Outcomes, No Propensity Score

|  | CESD |  | Binge |  | Painkiller |  | Hard drugs |  | Suicide |  |
| --- | --- | --- | --- | --- | --- | --- | --- | --- | --- | --- |
| College | -0.61^***^ | [-0.69,-0.52] | -0.07^***^ | [-0.09,-0.06] | -0.04^***^ | [-0.05,-0.04] | -0.01^***^ | [-0.02,-0.01] | -0.03^***^ | [-0.04,-0.02] |
| Age | -0.01 | [-0.03,0.02] | -0.00^**^ | [-0.01,-0.00] | 0.00 | [-0.00,0.00] | -0.00^***^ | [-0.01,-0.00] | -0.00^**^ | [-0.01,-0.00] |
| Male | -0.24^***^ | [-0.32,-0.15] | 0.05^***^ | [0.04,0.07] | -0.00 | [-0.01,0.01] | 0.02^***^ | [0.01,0.02] | -0.00 | [-0.01,0.01] |
| Hispanic | -0.13^*^ | [-0.25,-0.01] | 0.00 | [-0.01,0.02] | -0.01 | [-0.02,0.00] | 0.01 | [-0.00,0.01] | -0.01^*^ | [-0.03,-0.00] |
| Black(nonHisp) | 0.11^*^ | [0.00,0.21] | -0.04^***^ | [-0.06,-0.03] | 0.01 | [-0.01,0.02] | -0.01^*^ | [-0.02,-0.00] | 0.00 | [-0.01,0.01] |
| Asian(nonHisp) | -0.14 | [-0.32,0.03] | -0.04^**^ | [-0.07,-0.01] | -0.00 | [-0.02,0.02] | 0.02^*^ | [0.00,0.03] | -0.01 | [-0.03,0.01] |
| Other(nonHisp) | -0.33 | [-0.81,0.15] | -0.06 | [-0.13,0.02] | 0.00 | [-0.05,0.05] | 0.05^**^ | [0.02,0.09] | -0.02 | [-0.07,0.03] |
| Intercept | 6.73^***^ | [6.36,7.09] | 0.24^***^ | [0.18,0.29] | 0.06^**^ | [0.02,0.10] | 0.09^***^ | [0.07,0.12] | 0.14^***^ | [0.11,0.18] |
| AIC | 54444.23 |  | 8975.46 |  | 1082.69 |  | -7565.51 |  | 457.35 |  |
| BIC | 54503.42 |  | 9034.77 |  | 1141.96 |  | -7506.29 |  | 516.49 |  |
| Adj. R^2^ | 0.019 |  | 0.021 |  | 0.007 |  | 0.007 |  | 0.004 |  |
| Observations | 6145 |  | 6145 |  | 6145 |  | 6145 |  | 6145 |  |

Notes: 95% confidence intervals in brackets. ^*^ *p* < 0.05, ^**^ *p* < 0.01, ^***^ *p* < 0.001. *Source:* Add Health.

Table S8: Homogeneous Effect of College Completion on Selected Outcomes, With Propensity Score

|  | CESD |  | Binge |  | Painkillers |  | Hard drugs |  | Suicide |  |
| --- | --- | --- | --- | --- | --- | --- | --- | --- | --- | --- |
| College | -0.31^***^ | [-0.44,-0.18] | -0.05^***^ | [-0.07,-0.03] | -0.03^***^ | [-0.04,-0.01] | -0.01^**^ | [-0.02,-0.00] | -0.02^*^ | [-0.03,-0.00] |
| Propensity score | -1.20^***^ | [-1.48,-0.91] | -0.08^***^ | [-0.13,-0.04] | -0.10^***^ | [-0.13,-0.06] | -0.00 | [-0.03,0.02] | -0.06^***^ | [-0.09,-0.03] |
| Intercept | 7.31^***^ | [6.80,7.83] | 0.27^***^ | [0.19,0.35] | 0.12^***^ | [0.06,0.17] | 0.09^***^ | [0.05,0.13] | 0.15^***^ | [0.09,0.20] |
| AIC | 27346.4 |  | 4561.32 |  | 450.91 |  | -3856.39 |  | 295.10 |  |
| BIC | 27406.9 |  | 4621.90 |  | 511.47 |  | -3795.89 |  | 355.68 |  |
| Adj. R^2^ | 0.028 |  | 0.024 |  | 0.014 |  | 0.005 |  | 0.007 |  |
| Observations | 6145 |  | 6145 |  | 6145 |  | 6145 |  | 6145 |  |

Notes: 95% confidence intervals in brackets. ^*^ *p* < 0.05, ^**^ *p* < 0.01, ^***^ *p* < 0.001. Modelling approach as in (Xie et al., 2012). *Source:* Add Health.

Table S9: Homogeneous Effect of College Completion on Selected Outcomes, With Propensity Score in 10 Categories

|  | CESD |  | Binge |  | Painkiller |  | Hard drugs |  | Suicide |  |
| --- | --- | --- | --- | --- | --- | --- | --- | --- | --- | --- |
| College | -0.31^***^ | [-0.44,-0.18] | -0.05^***^ | [-0.07,-0.03] | -0.03^***^ | [-0.04,-0.01] | -0.01^**^ | [-0.02,-0.00] | -0.02^*^ | [-0.03,-0.00] |
| 1.pscorec10 | 0.58^***^ | [0.33,0.84] | 0.02 | [-0.02,0.06] | 0.05^***^ | [0.02,0.08] | 0.03^**^ | [0.01,0.05] | 0.01 | [-0.02,0.04] |
| 2.pscorec10 | 0.38^**^ | [0.13,0.64] | 0.03 | [-0.01,0.07] | 0.01 | [-0.02,0.03] | 0.01 | [-0.01,0.03] | 0.00 | [-0.03,0.03] |
| 3.pscorec10 | 0.12 | [-0.14,0.37] | 0.02 | [-0.02,0.06] | 0.00 | [-0.02,0.03] | -0.00 | [-0.02,0.02] | 0.01 | [-0.02,0.03] |
| 4.pscorec10 | 0.01 | [-0.24,0.27] | -0.01 | [-0.04,0.03] | -0.01 | [-0.04,0.02] | 0.01 | [-0.01,0.03] | -0.01 | [-0.04,0.01] |
| 5.pscorec10 | 0.00 | [0.00,0.00] | 0.00 | [0.00,0.00] | 0.00 | [0.00,0.00] | 0.00 | [0.00,0.00] | 0.00 | [0.00,0.00] |
| 6.pscorec10 | -0.03 | [-0.29,0.22] | -0.04 | [-0.07,0.00] | -0.00 | [-0.03,0.02] | 0.00 | [-0.02,0.02] | -0.02 | [-0.04,0.01] |
| 7.pscorec10 | -0.07 | [-0.33,0.18] | -0.04 | [-0.08,0.00] | -0.01 | [-0.04,0.02] | 0.01 | [-0.01,0.03] | -0.02 | [-0.05,0.00] |
| 8.pscorec10 | -0.21 | [-0.46,0.05] | -0.02 | [-0.06,0.02] | -0.03 | [-0.05,0.00] | 0.01 | [-0.01,0.03] | -0.03^*^ | [-0.06,-0.01] |
| 9.pscorec10 | -0.30^*^ | [-0.56,-0.04] | -0.04^*^ | [-0.08,-0.00] | -0.03^*^ | [-0.06,-0.00] | 0.01 | [-0.01,0.03] | -0.04^*^ | [-0.06,-0.01] |
| 10.pscorec10 | -0.20 | [-0.46,0.06] | -0.05^*^ | [-0.09,-0.01] | -0.02 | [-0.05,0.00] | 0.02^*^ | [0.00,0.05] | -0.03 | [-0.05,0.00] |
| Intercept | 6.33^***^ | [6.14,6.51] | 0.18^***^ | [0.15,0.21] | 0.09^***^ | [0.07,0.11] | 0.03^***^ | [0.01,0.04] | 0.09^***^ | [0.07,0.11] |
| AIC | 27382.88 |  | 4621.86 |  | 452.70 |  | -3841.00 |  | 304.44 |  |
| BIC | 27456.79 |  | 4695.89 |  | 526.71 |  | -3767.05 |  | 378.47 |  |
| Adj. R^2^ | 0.022 |  | 0.015 |  | 0.014 |  | 0.003 |  | 0.005 |  |
| Observations | 6145 |  | 6145 |  | 6145 |  | 6145 |  | 6145 |  |

Notes: 95% confidence intervals in brackets. ^*^ *p* < 0.05, ^**^ *p* < 0.01, ^***^ *p* < 0.001. Modelling approach as in (Xie et al., 2012). *Source:* Add Health.

Table S10: Means for the Five Outcomes By College Completion and Propensity Strata

| Propensity | Low | | Medium | | High | |
| --- | --- | --- | --- | --- | --- | --- |
| Finished college | Yes | No | Yes | No | Yes | No |
|  | Mean | Mean | Mean | Mean | Mean | Mean |
| CESD | 0.12 | 0.21 | 0.10 | 0.16 | 0.10 | 0.11 |
| Binge | 0.16 | 0.20 | 0.10 | 0.16 | 0.09 | 0.14 |
| Painkillers | 0.04 | 0.10 | 0.05 | 0.08 | 0.03 | 0.05 |
| Hard drugs | 0.00 | 0.04 | 0.02 | 0.03 | 0.03 | 0.03 |
| Suicide | 0.05 | 0.09 | 0.05 | 0.08 | 0.04 | 0.05 |

Notes: Number of observations 6145. *Source:* Add Health.
